# 27-Hydroxycholesterol contributes to cognitive deficits in APP/PS1 transgenic mice through microbiota dysbiosis and intestinal barrier dysfunction

**DOI:** 10.1186/s12974-020-01873-7

**Published:** 2020-06-27

**Authors:** Ying Wang, Yu An, Weiwei Ma, Huiyan Yu, Yanhui Lu, Xiaona Zhang, Yushan Wang, Wen Liu, Tao Wang, Rong Xiao

**Affiliations:** 1 School of Public Health, Beijing Key Laboratory of Environmental Toxicology, Capital Medical University, No.10 Xitoutiao, You An Men Wai, Beijing, 100069 China; 2 School of Nursing, Peking University, Beijing, China

**Keywords:** 27-Hydroxycholesterol, Alzheimer’s disease, Gut microbiota, Intestinal barrier dysfunction, Inflammation, Cognitive deficits

## Abstract

**Background:**

Research on the brain-gut-microbiota axis has led to accumulating interest in gut microbiota dysbiosis and intestinal barrier dysfunction in Alzheimer’s disease (AD). Our previous studies have demonstrated neurotoxic effects of 27-hydroxycholesterol (27-OHC) in in vitro and in vivo models. Here, alterations in the gut microbiota and intestinal barrier functions were investigated as the possible causes of cognitive deficits induced by 27-OHC treatment.

**Methods:**

Male APP/PS1 transgenic and C57BL/6J mice were treated for 3 weeks with 27-OHC (5.5 mg/kg/day, subcutaneous injection) and either a 27-OHC synthetase inhibitor (anastrozole, ANS) or saline. The Morris water maze and passive avoidance test were used to assess cognitive impairment. Injuries of the intestine were evaluated by histopathological examination. Intestinal barrier function was assessed by plasma diamine oxidase (DAO) activity and d-lactate. Systemic and intestinal inflammation were evaluated by IL-1β, TNF-α, IL-10, and IL-17 concentrations as determined by ELISA. The fecal microbiome and short-chain fatty acids (SCFAs) were analyzed using 16S rDNA sequencing and ultra-performance liquid chromatography-mass spectrometry (UPLC-MS). Tight junction proteins were evaluated in the ileum and colon by qRT-PCR and Western blots. Tight junction ultrastructure was examined by transmission electron microscopy.

**Results:**

Treatment with 27-OHC resulted in severe pathologies in the ileum and colon. There was impaired intestinal barrier integrity as indicated by dilated tight junctions and downregulation of tight junction proteins, including occludin, claudin 1, claudin 5, and ZO-1, and signs of inflammation (increased IL-1β, TNF-α, and IL-17). Fecal 16S rDNA sequencing and taxonomic analysis further revealed a decreased abundance of *Roseburia* and reduced fecal levels of several SCFAs in 27-OHC-treated mice. Meanwhile, co-treatment with ANS reduced intestinal inflammation and partially preserved intestinal barrier integrity in the presence of 27-OHC.

**Conclusions:**

The current study demonstrates for the first time that 27-OHC treatment aggravates AD-associated pathophysiological alterations, specifically gut microbiota dysbiosis and intestinal barrier dysfunction, which suggests that the gut microbiome and intestinal barrier function warrant further investigation as potential targets to mitigate the neurotoxic impact of 27-OHC on cognitive function and the development of AD.

## Introduction

Alzheimer’s disease (AD) is a highly prevalent neurodegenerative disease in older people. It is clinically characterized by progressive cognitive impairment and irreversible impairment of the ability to perform activities of daily living [1]. It is pathologically characterized by extracellular amyloid-β (Aβ) deposition in senile plaques and the intracellular presence of neurofibrillary tangles comprised of abnormally hyperphosphorylated tau protein [2]. As a multifactorial disease resulting from a combination of genetic and environmental risk factors, so far AD has neither a cure nor any treatment. Since an estimated 131.5 million patients are predicted to be diagnosed with AD by 2050 [3], exploration of its pathogenesis and treatment strategies are essential to provide insight into the etiology of the disease and to develop therapeutic and preventive strategies.

Alterations in gut microbiota have been reported to be intimately involved in the pathogenesis of AD. Human studies have reported that the distinct gut microbiota of AD patients is characterized by enriched in Enterobacteriaceae as well as abundant Bacteroides, Actinobacteria, and Selenomonadales as compared with predementia stage mild cognitive impairment (MCI) or healthy controls [4]. Moreover, Bauerl et al. [5] have demonstrated how the gut microbiota shifts during aging and the links between perturbations in the microbiome to AD pathology in the transgenic APP/PS1 (TG) mouse model, a well-established deterministic mouse model of AD. Recent human and animal studies have shown inflammation-related bacterial profiles with (neuro)inflammation proposed as an etiological link [6].

The bidirectional networks between the brain and gut microbiota are maintained through metabolic, immune, endocrine, and neuronal pathways [7]. Recent evidence has suggested a major role of gut microbiota in various neurological conditions mediated by peripheral and central immune activation and inflammation. Altered gut microbiota and blood pro-inflammatory cytokine profiles were concurrently exhibited in patients with cognitive impairment and brain amyloidosis [8]. It is now well established that inflammation, activated by central stimuli (such as Aβ) and peripheral stimuli (such as endotoxins, lipopolysaccharide), plays an important role in AD pathogenesis [9].

On the one hand, the intestinal barrier permeability may be altered by endogenous or exogenous factors as an outcome of inflammatory processes, which may cause the introduction of undesired pathogens into the body; on the other hand, neuroinflammatory conditions are associated with blood-brain barrier (BBB) disruption, which may fail to maintain the permeability and ensure homeostasis of the central nervous system (CNS) [10]. Consequently, peripheral and central inflammatory processes contribute to a pro-inflammatory gut environment, a disturbed intestinal barrier and BBB permeability, and impaired brain function in the elderly, which in turn enhances the progression of AD.

Despite being relatively stable, the gut microbiota composition gradually undergoes changes due to lifestyle, geographical location, and dietary habits [11]. An increasing number of studies have demonstrated pro-inflammatory characteristics of cholesterol oxidation products, namely oxysterols, in human pathophysiology, especially side chain oxysterols with enzymatic origins [12]. As one of the major side chains and enzymatic origin oxysterols found in the human circulation, 27-hydroxycholesterol (27-OHC) is ubiquitously produced by the mitochondrial enzyme CYP27A1 [13].

27-OHC has been suggested to be involved in the pathogenesis of gastrointestinal and neurodegenerative diseases [14] due to its strong pro-inflammatory properties and ability to act as an endogenous selective estrogen receptor modulator (SERM). It is able to act as ligands for different isoforms of the estrogen receptor (ER) [15]. Rossin et al. [16] have reported increased production of 27-OHC in advanced stage human colorectal cancer and it may contribute to cancer cell survival and infiltration.

Our recent study [17] demonstrated that 27-OHC may cause inflammatory damage to neurons and astrocytes by activating the TGF-β/NF-κB or TLR4/TGF-β signaling pathways, resulting in the subsequent release of inflammatory cytokines. Brooks et al. also showed higher serum 27-OHC modifies estrogen receptor (ER) expression and increases neurodegeneration in the rabbit hippocampus [18]. Moreover, in breast tumors, 27-OHC serves as a partial ER agonist and can stimulate tumor growth [19].

Anastrozole (ANS), an anti-breast cancer pharmaceutical, was tested previously in mice in vivo by Mast et al. [20] and was identified as a strong CYP27A1 inhibitor, as subcutaneous injection of ANS caused significant decreases in plasma and hepatic 27-OHC. Consequently, anastrozole could be a therapeutic option for the treatment of ER-positive breast cancer. Our previous in vivo studies have also demonstrated that the use of ANS decreased the plasma and brain levels of 27-OHC and reversed the neurotoxicity induced by 27-OHC [21, 22].

However, as reported by the aforementioned articles, some findings of the in vivo studies could not be replicated in differentiated CaCo-2 cells, human neuroblastoma cells, or rat glioma cells. Therefore, the underlying mechanisms by which 27-OHC exerts its neurotoxic effects are still unclear, and whether 27-OHC can trigger cognitive deficits through inflammatory responses in the gut–brain axis has rarely been examined in vivo. Bearing this in mind, we considered it essential to extend our research from cell models to animal models.

The mutations in presenilin 1 and amyloid precursor protein (APP) genes are the most commonly recognized triggers of familial AD, which led to the development of the APP/PS1 transgenic mouse model that mimics some of the pathology in AD and presents with increased Aβ1-42 production [23]. In this study, we used the APP/PS1 transgenic mouse model to investigate the possible mechanisms associated with excessive 27-OHC that induce pro-inflammatory and neurotoxic effects. We propose that excessive circulating and brain 27-OHC triggers peripheral and central inflammatory processes and gut microbiota dysbiosis, coupled with increased intestinal barrier permeability, and eventually severe Aβ deposition in the APP/PS1 mice.

## Materials and methods

### Animals and treatments

Specific pathogen-free (SPF) male APP/PS1 transgenic and sex- and age-matched wild-type C57BL/6J mice, 6 months old, weighing 30–35 g, were purchased from Beijing Vital River Laboratory Animal Technology Co., Ltd. (Beijing, China; certification number YXK (Beijing) 2017-0022). The mice were housed in standard individual ventilated cages in a temperature- (22–25 °C) and humidity- (50–60%) controlled room with a 12 h dark-light cycle. Food and water were available ad libitum.

Our previous study has already investigated the effects of intravenous injection of 27-OHC in rats [24]. However, to date, there is no established dosage for subcutaneous injection of 27-OHC in C57BL/6J mice. Here, we conducted the first dose-finding study for subcutaneous administration of 27-OHC to C57BL/6J mice to pave the way for further studies investigating the effects of 27-OHC on the gut microbiota and intestinal barrier in APP/PS1 transgenic mice.

The 27-OHC doses used for the current study were based on the known effective dose in rats (70 μM/day) [24], the physiological levels of 27-OHC in the blood (0.15–0.73 μM) [25], and that taken up by the brain per 5 mg/24 h [26]. The whole blood volume of mice is 7.6 mL/100 g. Thus, a dose of approximately 5.5 mg/g would be effective if assuming direct translatability from rats to mice. Nevertheless, since differences in metabolism can affect the actual effective dose, 27-OHC was tested in six different doses ranging around this value (i.e., 0.275, 0.55, 1.65, 3.3, 5.5, and 8.25 mg/kg). 27-OHC (10 mg, R&D Systems Co., Ltd., USA) was dissolved in 1 mL ethanol and 9 mL 0.9% saline and then diluted to 1 mg/mL before use. The C57BL/6J mice (*n* = 10/group) were randomly assigned to seven groups (i.e., 0.275, 0.55, 1.65, 3.3, 5.5, and 8.25 mg/kg 27-OHC/day or vehicle [0.9% saline]). After treatment for 3 weeks, behavioral tests were carried out.

Next, the APP/PS1 mice (6 months old, *n* = 10/group) and matched wild-type C57BL/6J mice (6 months old, *n* = 10/group) were randomly divided into five groups: group I: WT control (C57BL/6J mice); group II: APP/PS1 control; group III: WT mice treated with 27-OHC (selected effective dose for subcutaneous injection); group IV: APP/PS1 mice treated with 27-OHC (selected effective dose for subcutaneous injection); group V: APP/PS1 mice treated with anastrozole (ANS, an inhibitor of CYP27A1, Enzo Life Sciences, Inc., Switzerland, 0.2 mg/day, subcutaneous injection); group VI: WT mice cotreated with 27-OHC (selected effective dose for subcutaneous injection) plus ANS (0.2 mg/day, subcutaneous injection); and group VII: APP/PS1 mice cotreated with 27-OHC (selected effective dose for subcutaneous injection) plus ANS (0.2 mg/day, subcutaneous injection). The APP/PS1-control and C57BL/6J-control groups received the same volume of 0.9% normal saline alone according to the same schedule.

**Fig. 1:**
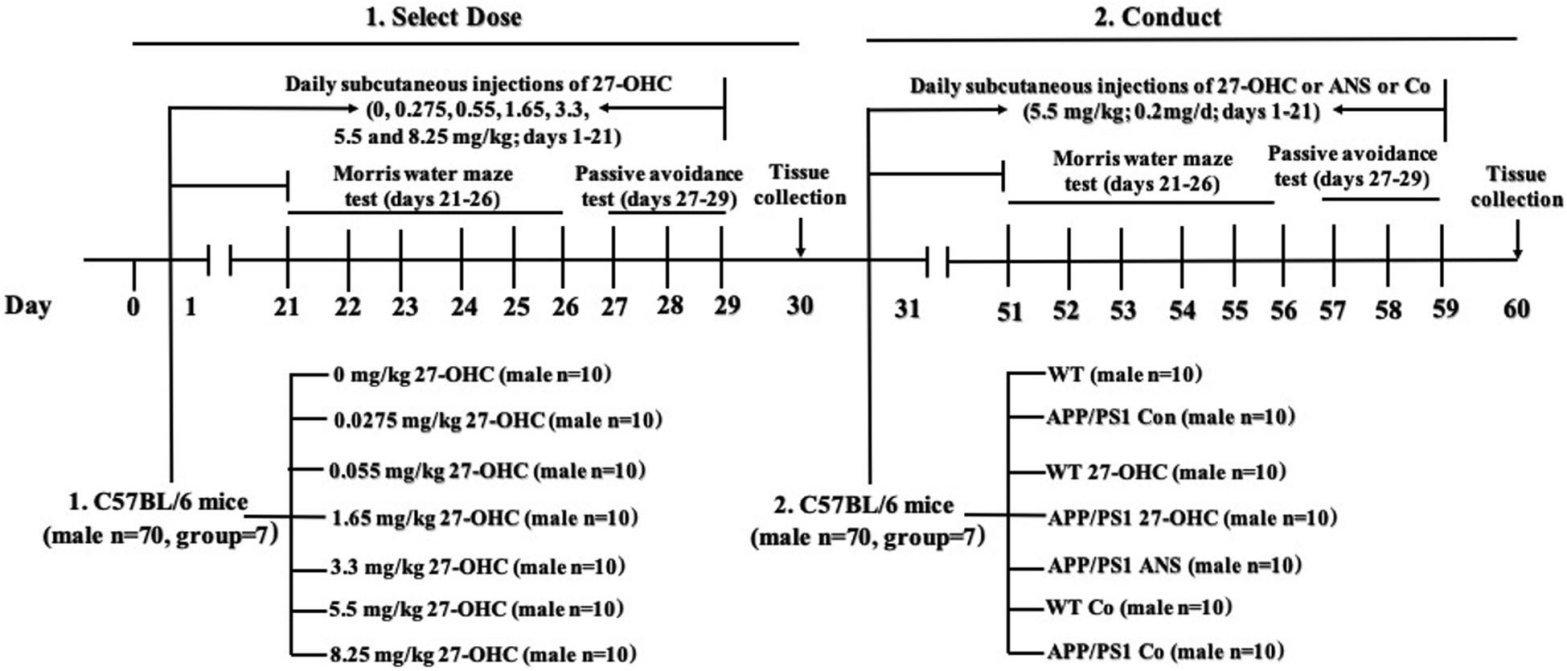
A schematic diagram of drug treatment and protocol design, *n* = 10/group

The Morris water maze and passive avoidance test were used to evaluate their learning and memory deficits. The experimental schedule is shown in Fig. 1. All experimental procedures were conducted in accordance with the National Institutes of Health Guide for the Care and Use of Laboratory Animals (NIH Publications No. 8023, revised 1978) and were approved by the ethics committee of Capital Medical University (AEEI-2014-047).

### Morris water maze

Spatial learning and memory ability were evaluated by the Morris water maze test, which consists of orientation navigation and spatial probe tests. The test was performed in a circular tank (120 cm in diameter, 50 cm height) divided into four quadrants. A hidden escape platform (9 cm in diameter) was placed 2.0 cm below the water in the center of one quadrant. The water was colored with titanium dioxide for the C57BL/6J and APP/PS1 mice and adjusted to 22 ± 1 °C. The tank was placed in a dimly lit and soundproof room with various visual cues.

The orientation navigation test was performed for five consecutive days, and the mice were released into the water from different starting positions and allowed to swim freely until they reached the hidden platform. This was repeated four times each day with an interval of 30 min. The latency to reach the platform and swimming trajectory were recorded by a computer-controlled video tracking system (Morris water maze video analysis system, Institute of Materia Medica, Chinese Academy of Medical Sciences, China) for a maximum duration of 90 s. Mice that failed to reach the hidden platform within 90 s were placed on it for 15 s. The escape latency was determined as the time from the starting position to the platform. Afterwards, the spatial probe test was carried out. The platform was removed on the sixth day. The mice were released from a position opposite from where the platform used to be and allowed to swim for 90 s in the maze. The time spent in the target quadrant, the time spent crossing where the platform was originally located, swimming speed, and swimming trajectory were recorded.

### Passive avoidance test

The passive avoidance test is a fear-motivated test used to evaluate learning and retention memory. It is conducted in an apparatus that consists of two identical compartments (light and dark) 25 × 25 × 25 cm in size with a sliding door and grid-metal floor. The test enables mice to learn to restrain their innate behavior through moving from the illuminated compartment to the dark compartment. During the passive avoidance preacquisition trial, the mice were placed into the light compartment for a 5-min adaptation. The sliding door was automatically closed as soon as the mouse completely entered the dark compartment. Subsequently, the mouse was subjected to an electrical foot-shock (0.5 mA for 3 s) via the floor grid. The latency to enter the dark compartment was detected and analyzed and mice with latencies longer than 300 s were omitted from the study.

The retention memory test trial was tested 48 h after the acquired response had been established by recording their latency to enter the dark compartment. The latency to enter the dark compartment and frequency of entries into the dark compartment were recorded and directly linked to the retention memory. A greater latency and lower frequency of entry indicates better memory.

### Blood and tissue harvesting for biochemical analysis

APP/PS1 and C57BL/6J mice were anesthetized with 5% chloral hydrate (Beijing Solarbio Life Sciences Co., Ltd., China). Fasting blood was drawn via cardiac puncture with a syringe. After being centrifuged at 3000 g for 15 min at 4 °C, serum and ethylenediaminetetraacetic acid (EDTA)-plasma samples were collected and stored at − 80 °C until use. Following blood collection, the mice were perfused transcardially with ice-cold 0.2 M phosphate buffer solution (PBS). Their brains and the entire intestine were removed and fixed in 4% paraformaldehyde at 4 °C followed by processing for paraffin embedding and sectioning for histopathological analysis. Meanwhile, at necropsy, tissue samples from the brain, liver, kidneys, spleen, and intestine that showed gross changes were examined. The organ coefficient is expressed as the ratio to body weight to account for differences in organ weight-related effects and overt signs of toxicity.

### Histopathological examination

A part of the fixed brain as well as the distal ileum and proximal colon tissues were embedded in paraffin, cut into 5 μm sections, stained with hematoxylin and eosin (HE), and examined by light microscopy (*n* = 6/group). The Aβ load in the brains was assessed using Bielschowsky silver stain (BSS), which was performed according to the previously published protocols [27]. A portion of intestinal (distal ileum specimens and proximal colon) tissue was processed for ultrastructural examination. After being fixed, dehydrated, dried, and stained, the intestinal sections were examined by a JEM-2100 transmission electron microscope (TEM, Tokyo, Japan).

### Plasma and brain 27-OHC levels

High-performance liquid chromatography-mass spectrometry (Agilent Technologies Inc., USA) was used to measure the plasma and brain levels of 27-OHC (*n* = 6/group) according to our previously reported protocol [22].

### Plasma and brain Aβ measurement by ELISA

The levels of Aβ1-40 and Aβ1-42 in the plasma and brains were determined by ELISA kits (Thermo Fisher Scientific Co., Ltd., USA) in accordance with the manufacturer’s instructions (*n* = 6/group). Each experimental sample was run in triplicate. The data were recorded at 450 nm using a microplate reader (Bio-Rad Laboratories Co., Ltd., USA).

### Plasma and liver cholesterol levels measurement by ELISA

Plasma and liver levels of total cholesterol (TC), triglyceride (TG), high-density lipoprotein cholesterol (HDL-C), and low-density lipoprotein cholesterol (LDL-C) were measured by commercial kits (Jiancheng Bioengineering Institute, Nanjing, China). All of the procedures were performed according to the manufacturers’ protocols (*n* = 6/group).

### Plasma cytokine analysis

Levels of IL-1β, IL-10, TNF-α, and IL-17 in the plasma were measured by enzyme-linked immunosorbent assay ELISA kits (Thermo Fisher Scientific Co., Ltd., USA) following the manufacturers' instructions (*n* = 6/group).

### Assessment of intestinal barrier dysfunction and inflammation

Intestinal permeability (DAO and D-lactate) and tight junction proteins (occludin, claudin 1, claudin 5, and ZO-1) as well as intestinal inflammatory cytokines (IL-1β and IL-10) were measured to assess intestinal barrier dysfunction.

DAO activity and d-lactate in serum were measured by commercial kits (Jiancheng Bioengineering Institute, Nanjing, China). All of the procedures were performed according to the manufacturers’ protocols (*n* = 6/group).

The mRNA and protein expression of occludin, claudin 1 (CLOD-1), claudin 5 (CLOD-5), and ZO-1 in the ileum and colon were measured by quantitative real-time polymerase chain reaction (qRT-PCR, *n* = 6/group) and Western blots (*n* = 6/group). RNA extraction was performed according to the manufacturer’s instructions using the SV Total RNA Isolation System (Promega Corporation Co., Ltd., USA). The RNA concentration was measured and 1 μg of RNA was reverse-transcribed using a RevertAid First Strand cDNA Synthesis kit (Thermo Fisher Scientific Co., Ltd., USA) according to the manufacturer’s instructions. For relative quantification of mRNA levels, qRT-PCR was performed on a Real-time PCR Detection System (Bio-Rad Laboratories Co., Ltd., USA). All samples were measured in triplicate. The housekeeping gene glyceraldehyde 3-phosphate dehydrogenase (GAPDH) served as the reference for standardization. Quantitative measurements of target gene levels relative to controls were performed with the 2-ΔΔCt method.

For the Western blots, intestinal tissues were lysed in freshly prepared RIPA buffer (50 mM Tris buffer saline, 0.5% deoxysodium cholate, 1 mM EDTA, 150 mM NaCl, 1% NP-40, and 1 mM PMSF). The concentrations of the protein extracts were determined by a BCA assay kit (Beyotime Biotechnology Co., Ltd., China). Proteins (50 μg per lane) were separated by sodium dodecyl sulfate-polyacrylamide gel by electrophoresis (SDS-PAGE) and transferred onto PVDF membranes (Merck Millipore Co., Ltd., Germany). The membranes were blocked with 5% bovine serum albumin prepared in TBS (Tris-buffered saline, pH 7.5 containing 0.1% Tween-20) for 1 h at room temperature, then incubated with the primary antibodies overnight at 4 °C, followed by the HRP-conjugated secondary antibodies (1:5,000. Abcam Company Co., Ltd., USA). All primary antibodies were as follows: anti-ZO-1 (1:5000; Abcam Company Co., Ltd., USA), anti-Occludin (1:50,000; Abcam Company Co., Ltd., USA), anti-Cldn-1 (1:5000; Abcam Company Co., Ltd., USA), anti-Cldn-5 (1:5000; Abcam Company Co., Ltd., USA), and β-actin (1:5000; Abcam Company Co., Ltd., USA). Protein bands were visualized by an enhanced chemiluminescence kit (Pierce Protein Biology Co., Ltd., USA). The density of each band was measured using Image System Fusion FX (Vilber Lourmat Co., Ltd., France) and normalized to β-actin levels. The experiments were repeated three times. The plots for Western blot were individual samples representative for each group.

Levels of IL-1β, IL-10, TNF-α, and IL-17 in the distal ileum specimens and proximal colon were also measured by ELISA kits (Thermo Fisher Scientific Co., Ltd., USA) following the manufacturers’ instructions (*n* = 6/group).

### Fecal 16S rDNA sequencing

At the end of the treatment, fecal samples were collected (*n* = 6/group) and subjected to DNA isolation using a Bacterial DNA Kit (Omega, Shanghai, China). The DNA concentration was determined using a Nanodrop 1000 (Thermo Fisher Scientific, Wilmington, DE, USA). The 16S rDNA gene library preparation was conducted by using PCR amplification of the V3–V4 region. All libraries were sequenced using an Illumina HiSeq2500 platform (Illumina, San Diego, California, USA) at Biomarker Technologies, Beijing, China. Bioinformatic analyses were performed to compare the differences in the gut microbiota between the indicated experimental groups.

### Measurement of short-chain fatty acids

Fecal samples (50 mg) were mixed with an aliquot of 1000 μL of methanol/water (v/v = 1:1) mixture and then vortexed for 1 h and centrifuged at 132,000 r/min for 10 min. Then, 50 μL supernatant was transferred into a 1.5 mL centrifuge tube and mixed with 50 μL of propionic acid-d2 (1 μg/mL), which acts as an internal standard. Samples were then derivatized with 3NPH and centrifuged at 132,000 r/min for 10 min, and the supernatant was collected. An aliquot (5 μL) of the resulting derivatized material was injected into an ultra-performance liquid chromatograph (Waters UPLC I-Class) coupled to a tandem quadrupole mass spectrometer detector (Waters XEVO TQ-S Micro). Standard stock solutions of acetate, propionate, isobutyrate, butyrate, valerate, isovalerate, caproate, and heptanoic acid (ca. 1 mg/mL) were prepared by dissolving specific amounts of authentic standards in chloroform-methanol (1:1, v/v). The working standard solutions of the short-chain fatty acids (SCFAs) were serially diluted with chloroform-methanol (3:1, v/v) to obtain the concentrations needed for calibration curve standards (2, 10, 50, 200, 500, 1000 ng/mL). The estimate of the relative concentrations was based on the ratio of the area under the curve of the internal standard for each SCFA (*n* = 6/group).

### Statistical analysis

The distribution of the variables was first assessed by the Kolmogorov-Smirnov (K-S) test. If the data followed a normal distribution, data are presented as the mean ± standard deviation and parametric tests (one-way ANOVA for three or more groups) were performed; for ANOVA, least significance difference (LSD) testing was carried out for post hoc analysis. If the data were not normally distributed, the data are presented as the median (interquartile range) and nonparametric tests (Kruskal-Wallis test with Dunn’s post hoc analysis for three or more groups) were used by applying SPSS 19.0 software (Chicago, IL, USA) and Prism 6.0 (GraphPad Software, Inc., USA). Repeated measures analysis of variance (ANOVA) were performed for the water maze data as appropriate. Correlations were analyzed by Spearman’s test. *P* < 0.05 was considered statistically significant.

For 16S rDNA sequencing, sequences with ≥ 97% similarity were assigned to the same operational taxonomic units (OTUs). Representative sequences for each OTU were screened for further annotation. For each representative sequence, the Silva Database was used based on the mothur algorithm to annotate the taxonomic information. The following downstream data analyses were conducted in R software. Alpha diversity was applied for analyzing the complexity of species diversity for a sample through three indices, including Chao1, Shannon, and Ace. ANOVA was performed to evaluate α-diversity among the different groups. Beta diversity analysis was used to evaluate differences of the samples in species complexity. The linear discriminant analysis (LDA) effect size (LEfSe) method was performed to detect differentially abundant features with statistical significance and biological relevance among different groups and an LDA score of > 4 was used as the threshold. Metastats analysis was used to compare bacterial relative abundance between different groups.

## Results

### Establishing an effective dose for subcutaneous administration of 27-OHC in C57BL/6J mice

**Fig. 2:**
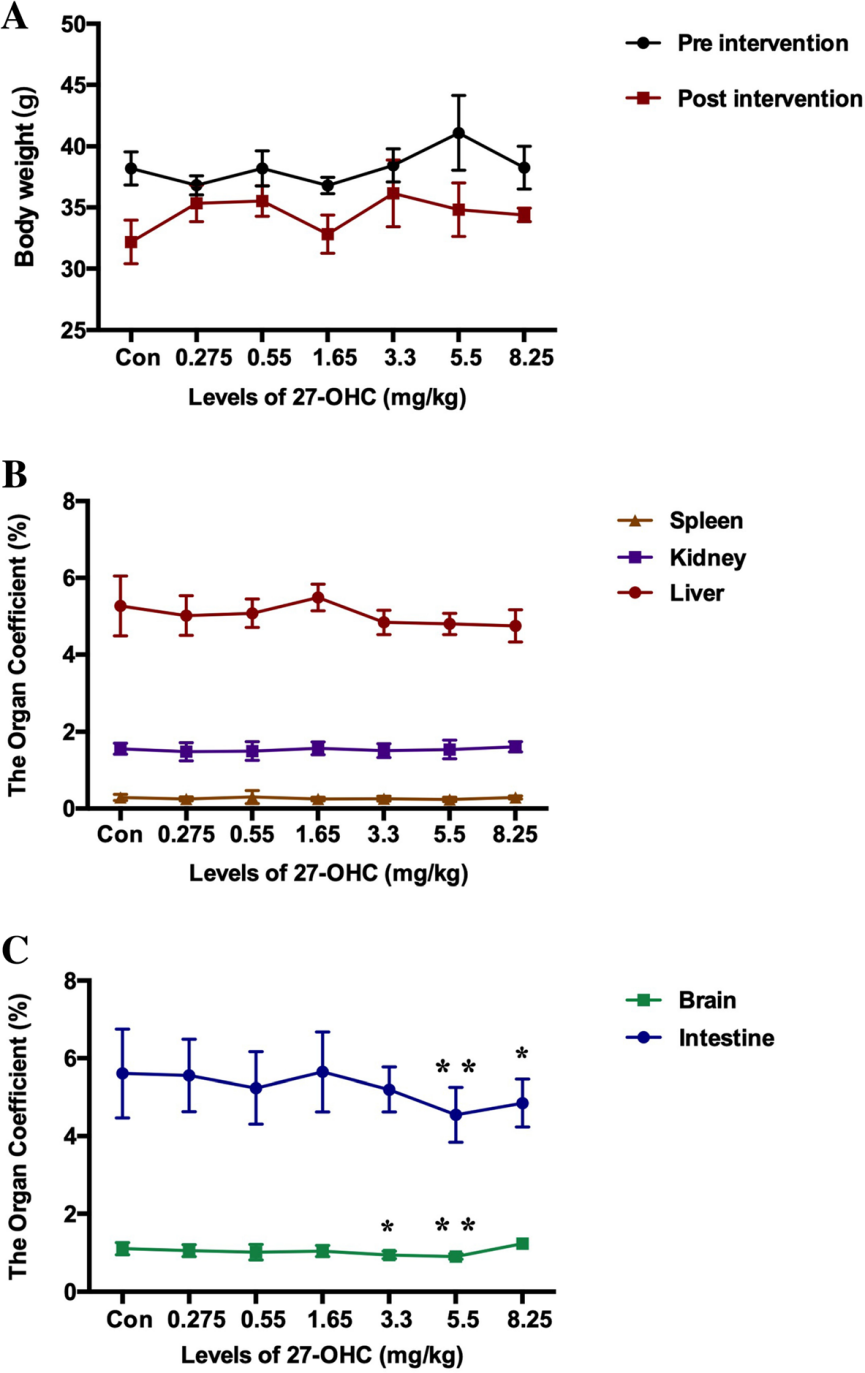
Comparison of body weight (**a**, mean ± SE) of C57BL/6J mice (*n* = 10/group) before and after subcutaneous administration of 27-OHC by different doses. Comparison of organ coefficient of spleen, kidney and liver (**b**) as well as brain and intestine (**c**) for different doses of 27-OHC treatment. One-way analysis of variance (ANOVA) was performed and post hoc comparisons were carried out using the LSD test. Asterisks indicate significant differences. *P < 0.05; **P < 0.01

**Fig. 3:**
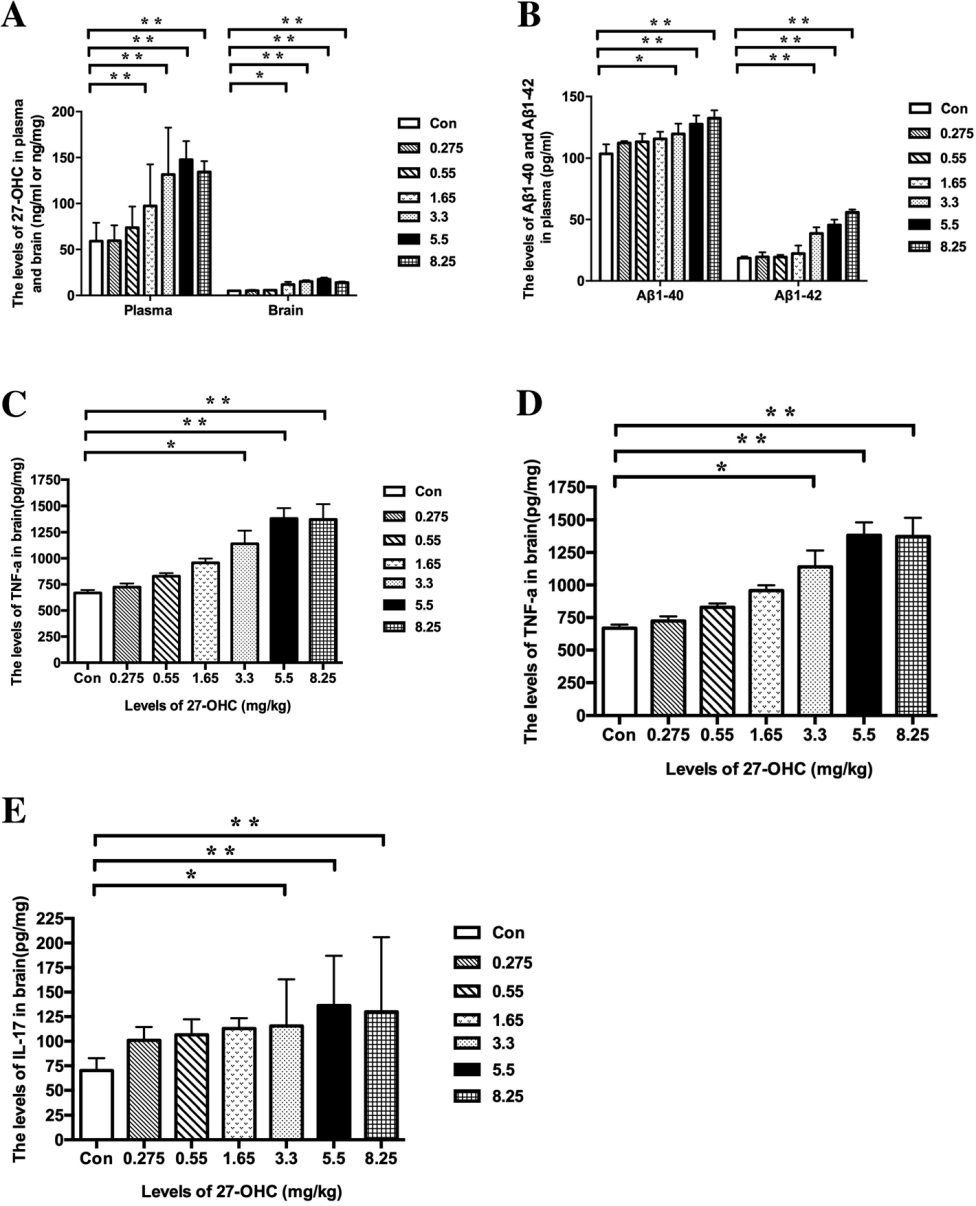
The plasma levels and brain levels of 27-OHC (**a**), plasma Aβ1-40 and Aβ1-42 (**b**), brain Aβ1-40 and Aβ1-42 (**c**), brain TNF-α (**d**), and IL-17 (**e**) in C57BL/6J mice for different doses of 27-OHC treatment. One-way analysis of variance (ANOVA) was performed and post hoc comparisons were carried out using the LSD test. Asterisks indicate significant differences. Data are presented as mean ± SEM. *n* = 10/group. *P < 0.05; **P < 0.01

The body weight of the treated C57BL/6J mice tended to decrease throughout the experimental period, albeit without significant differences (Fig. 2a). Moreover, 27-OHC treatment had no effect on spleen-to-body weight ratio, kidney-to-body weight ratio, or liver-to-body weight ratio at any of the doses (Fig. 2b). However, mice receiving 5.5 and 8.25 mg/kg 27-OHC displayed a significantly decreased brain-to-body weight ratio (*P* < 0.05, Fig. 2c) and mice receiving 3.3 and 5.5 mg/kg 27-OHC displayed a significantly decreased intestine-to-body weight ratio (*P* < 0.05, Fig. 2c) as compared to vehicle-treated mice. In addition, the plasma and brain levels of 27-OHC increased significantly in mice treated with doses of 27-OHC higher than 1.65 mg/kg (*P* < 0.05, Fig. 3a), which was accompanied by increased plasma and brain levels of Aβ (Aβ1-40 and Aβ1-42, *P* < 0.05, Fig. 3b, c) and brain levels of inflammatory cytokines (TNF-α and IL-17, *P* < 0.05, Fig. 3d, e) in mice treated with doses of 27-OHC higher than 3.3 mg/kg.

**Fig. 4:**
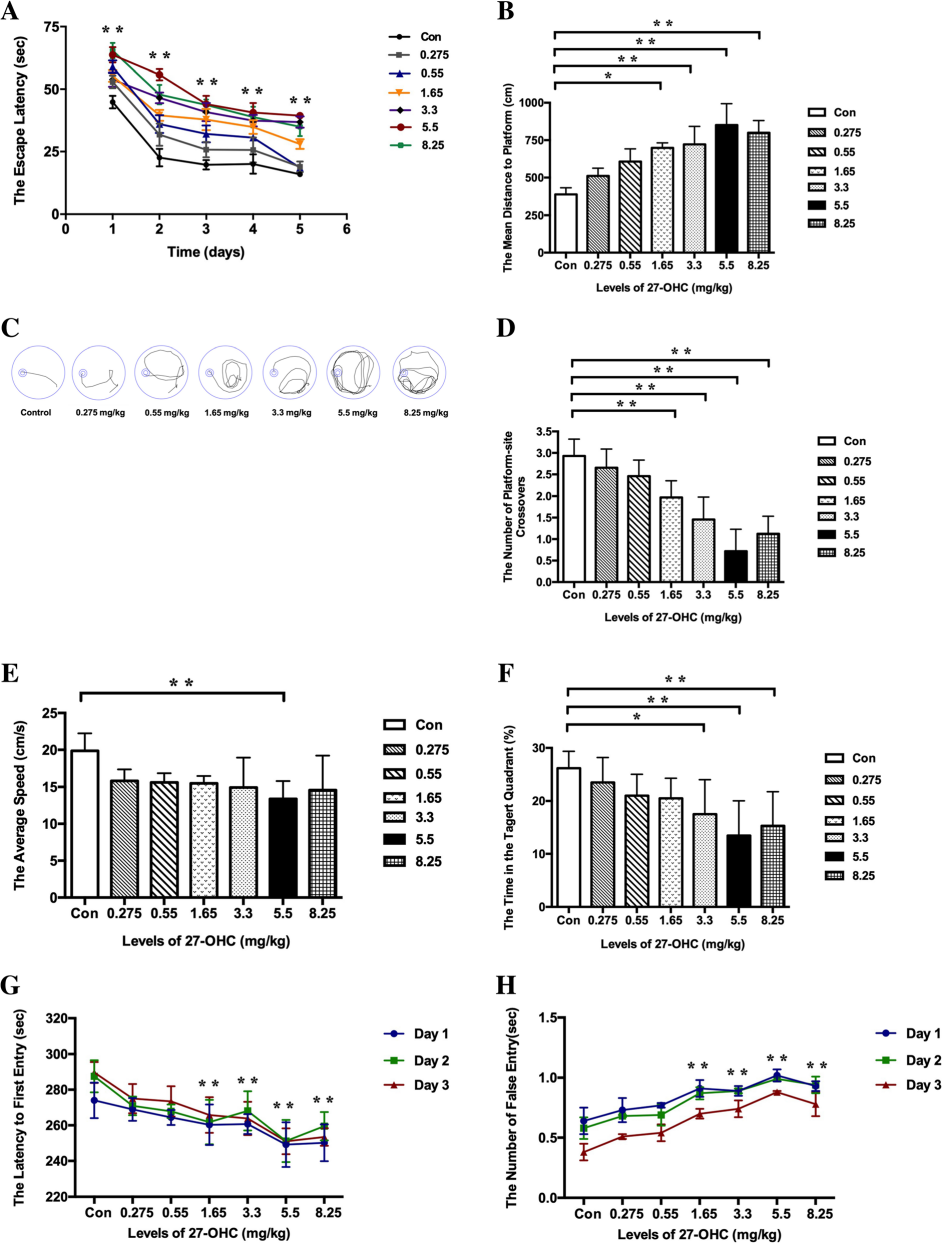
The escape latency (**a**), escape distance (**b**), and representative images of path (**c**) in orientation navigation test and the crossing-target number (**d**), swimming speed (**e**), and the target-quadrant abidance (**f**) in spatial probe test determined with the Morris water maze test as well as the latency to enter the dark area (**g**) and the frequency of entries to the dark area (**h**) determined with the passive avoidance test in C57BL/6J mice for different doses of 27-OHC treatment. One-way analysis of variance (ANOVA) was performed and post hoc comparisons were carried out using the LSD test. Asterisks indicate significant differences. Data are presented as mean ± SEM. *n* = 10/group. *P < 0.05; **P < 0.01

The Morris water maze and passive avoidance test were used to investigate whether subcutaneous injection of 27-OHC causes learning and memory deficits in mice. Regarding the Morris water maze, mice treated with doses of 27-OHC higher than 1.65 mg/kg displayed significant longer escape latency to the target platform (*P* < 0.05, Fig. 4a), shorter distances to the platform as shown by representative images of the path of travel (*P* < 0.05, Fig. 4b, c), and fewer crossing-target numbers (*P* < 0.05, Fig. 4d). With respect to the other parameters measured, 3 weeks of 5.5 mg/kg 27-OHC treatment resulted in a significant decrease in swimming speed (*P* < 0.05, Fig. 4e) and treatment with doses of 27-OHC higher than 3.3 mg/kg caused a significant decrease in target-quadrant abidance (*P* < 0.05, Fig. 4f).

In the passive avoidance test, the latency to enter the dark area (Fig. 4g) was significantly shortened and the frequency of entries into the dark compartment (Fig. 4h) was significantly increased in the mice receiving doses of 27-OHC higher than 1.65 mg/kg.

In summary, higher 27-OHC concentrations caused brain and intestine cell loss, Aβ deposits, neuroinflammation, and evident neurobehavioral impairment in mice. Only the 5.5 mg/kg dose of 27-OHC significantly affected all of the parameters measured in both the neurobehavioral tests and the neuropathologic examinations. Thus, the 5.5 mg/kg dosage can be used in further studies of 27-OHC in mice.

### 27-OHC treatment significantly affected plasma and brain 27-OHC levels, cholesterol levels, and Aβ load

**Fig. 5:**
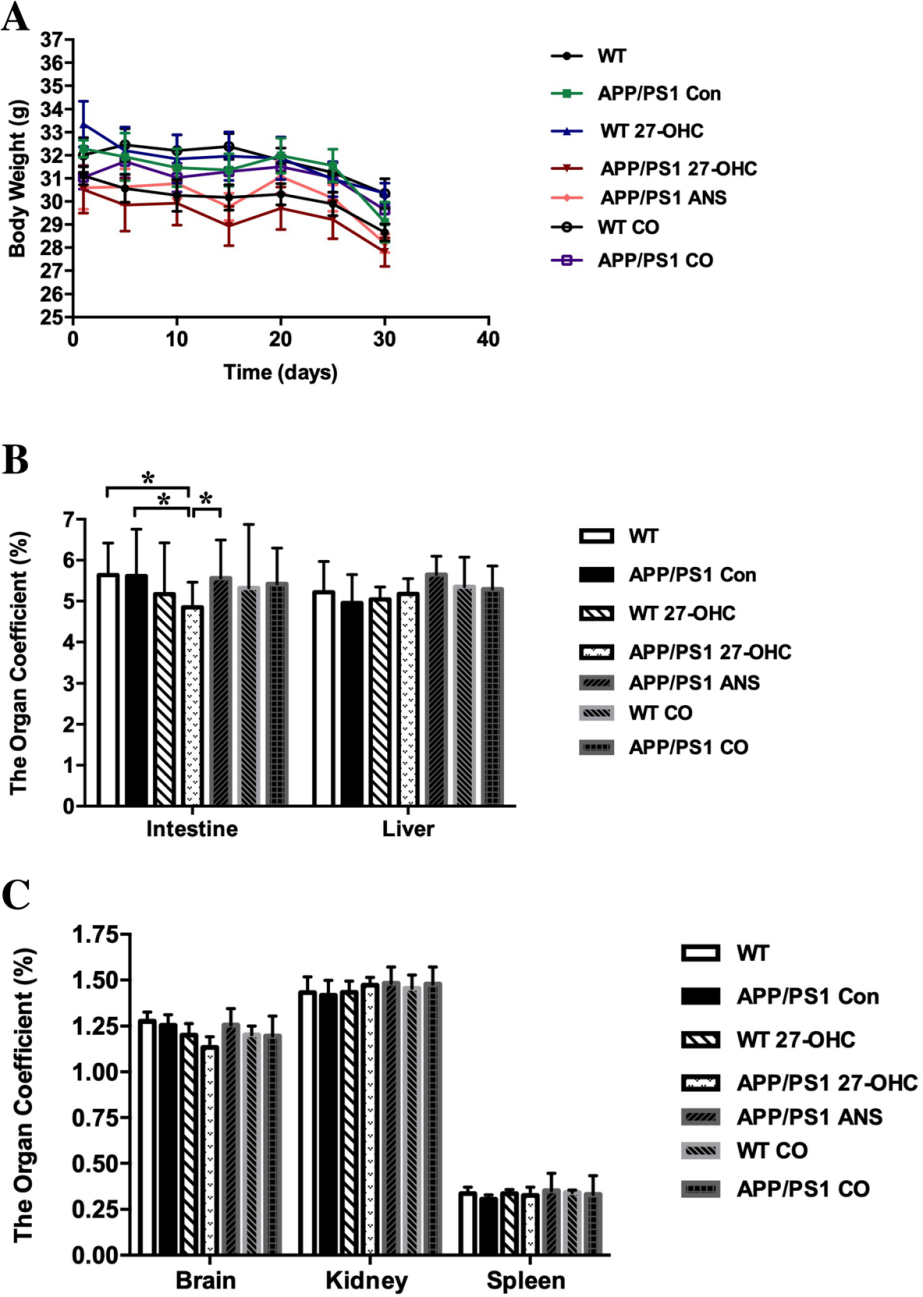
Comparison of body weight (**a**), organ coefficients of intestine and liver (**b**), as well as brain, kidney, and spleen (**c**) in different groups. Groups: WT: wild-type control group of C57BL/6J mice; APP/PS1 Con: transgenic control group of APP/PS1 mice; WT 27-OHC: C57BL/6J mice treated with 5.5 mg/kg 27-hydroxycholesterol; APP/PS1 27-OHC: APP/PS1 mice treated with 5.5 mg/kg 27-hydroxycholesterol; APP/PS1 ANS: APP/PS1 mice treated with 0.2 mg/day anastrozole; WT CO: C57BL/6J mice treated with 5.5 mg/kg 27-hydroxycholesterol plus 0.2 mg/day anastrozole; APP/PS1 CO: APP/PS1 mice treated with 5.5 mg/kg 27-hydroxycholesterol plus 0.2 mg/day anastrozole. Data are presented as mean ± SEM. One-way analysis of variance (ANOVA) was performed and post hoc comparisons were carried out using the LSD test. Asterisks indicate significant differences. *n* = 10/group. *P < 0.05; **P < 0.01

As illustrated in Fig. 5a, 27-OHC treatment tended to attenuate the body weight gain in WT and APP/PS1 mice, which were slightly lower than for control mice, ANS-treated, and 27-OHC plus ANS cotreated mice. However, 27-OHC treatment decreased the intestine-to-body weight ratio in APP/PS1 mice but did not affect other organ-to-body weight ratios (Fig. 5b, c). Treatment with ANS in the APP/PS1 groups could reverse the weight loss of the intestine (*P* < 0.05).

**Fig. 6:**
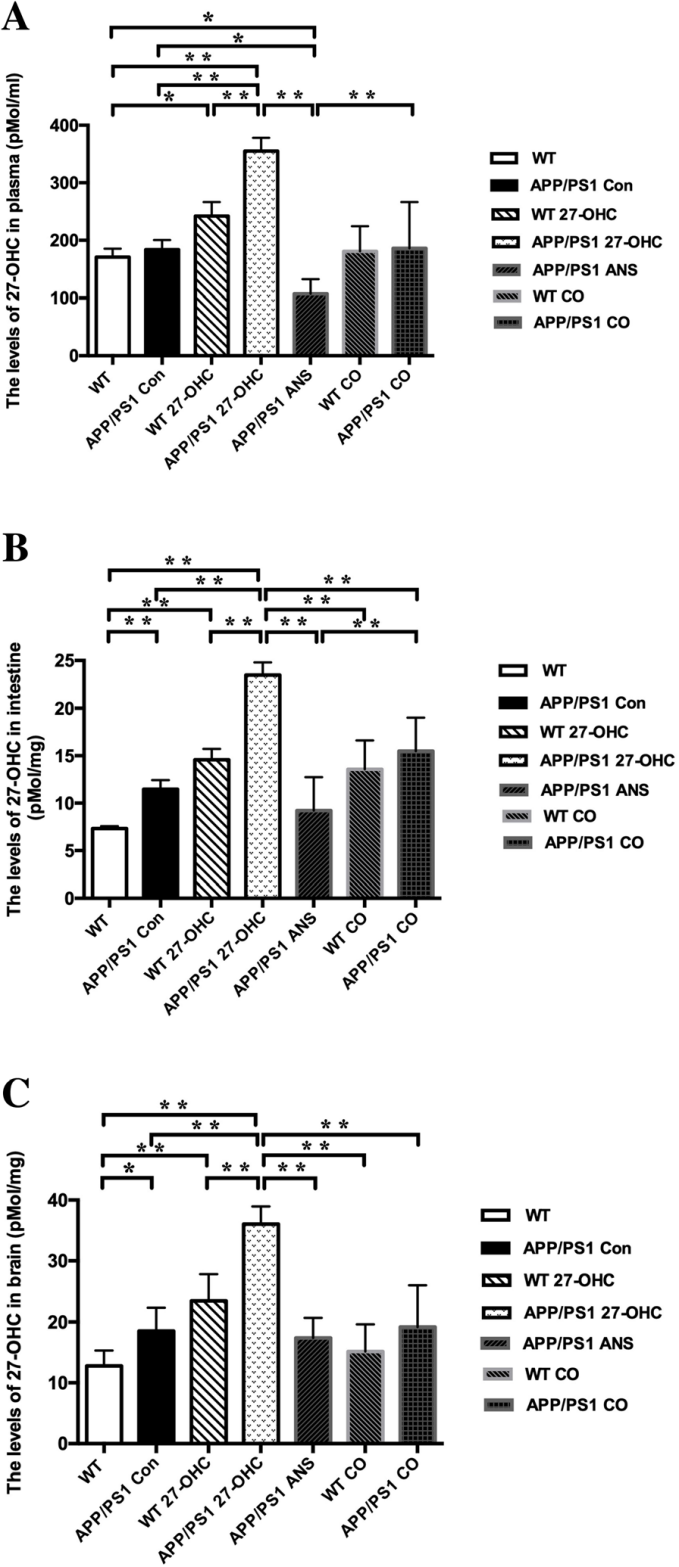
The plasma levels (**a**), intestine levels (**b**), and brain levels (**c**) of 27-OHC in different groups. Groups: WT: wild-type control group of C57BL/6J mice; APP/PS1 Con: transgenic control group of APP/PS1 mice; WT 27-OHC: C57BL/6J mice treated with 5.5 mg/kg 27-hydroxycholesterol; APP/PS1 27-OHC: APP/PS1 mice treated with 5.5 mg/kg 27-hydroxycholesterol; APP/PS1 ANS: APP/PS1 mice treated with 0.2 mg/day anastrozole; WT CO: C57BL/6J mice treated with 5.5 mg/kg 27-hydroxycholesterol plus 0.2 mg/day anastrozole; APP/PS1 CO: APP/PS1 mice treated with 5.5 mg/kg 27-hydroxycholesterol plus 0.2 mg/day anastrozole. One-way analysis of variance (ANOVA) was performed and post hoc comparisons were carried out using the LSD test. Asterisks indicate significant differences. Data are presented as mean ± SEM. *n* = 10/group. *P < 0.05; **P < 0.01

**Fig. 7:**
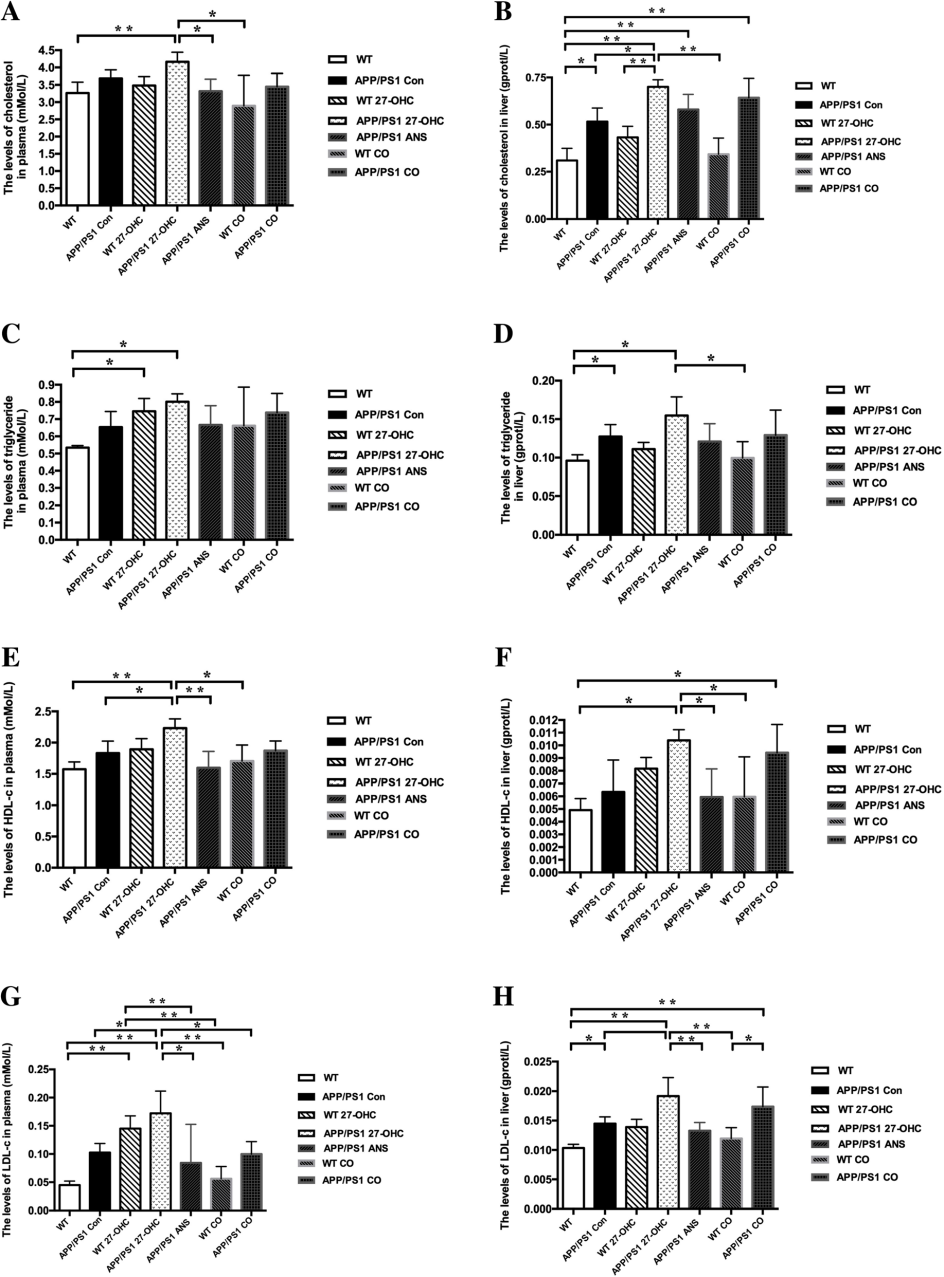
The levels of total cholesterol in plasma (**a**) and liver (**b**), triglyceride in plasma (**c**) and liver (**d**), HDL-C in plasma (**e**) and liver (**f**), as well as LDL-C in plasma (**g**) and liver (**h**) in different groups. Groups: WT: wild-type control group of C57BL/6J mice; APP/PS1 Con: transgenic control group of APP/PS1 mice; WT 27-OHC: C57BL/6J mice treated with 5.5 mg/kg 27-hydroxycholesterol; APP/PS1 27-OHC: APP/PS1 mice treated with 5.5 mg/kg 27-hydroxycholesterol; APP/PS1 ANS: APP/PS1 mice treated with 0.2 mg/day anastrozole; WT CO: C57BL/6J mice treated with 5.5 mg/kg 27-hydroxycholesterol plus 0.2 mg/day anastrozole; APP/PS1 CO: APP/PS1 mice treated with 5.5 mg/kg 27-hydroxycholesterol plus 0.2 mg/day anastrozole. One-way analysis of variance (ANOVA) was performed and post hoc comparisons were carried out using the LSD test. Asterisks indicate significant differences. Data are presented as mean ± SEM. *n* = 10/group. *P < 0.05; **P < 0.01. *HDL*-*C* high-density lipoprotein cholesterol, *LDL*-*C* low-density lipoprotein cholesterol

As shown in Fig. 6a–c, compared with the APP/PS1 and WT control group, 27-OHC treatment markedly increased, whereas ANS treatment significantly decreased the plasma, intestine, and brain levels of 27-OHC (*P* < 0.05). In addition, 27-OHC treatment could cause significant but different changes of plasma and liver cholesterol levels (Fig. 7a–h), such as increased liver TC and LDL-C in APP/PS1 mice, increased plasma TC and LDL-C in WT mice, and increased plasma HDL-C and LDL-C in APP/PS1 mice. On the other hand, ANS treatment could reverse the increases in plasma TC, HDL-C, and LDL-C as well as liver HDL-C and LDL-C induced by 27-OHC in APP/PS1 mice.

**Fig. 8:**
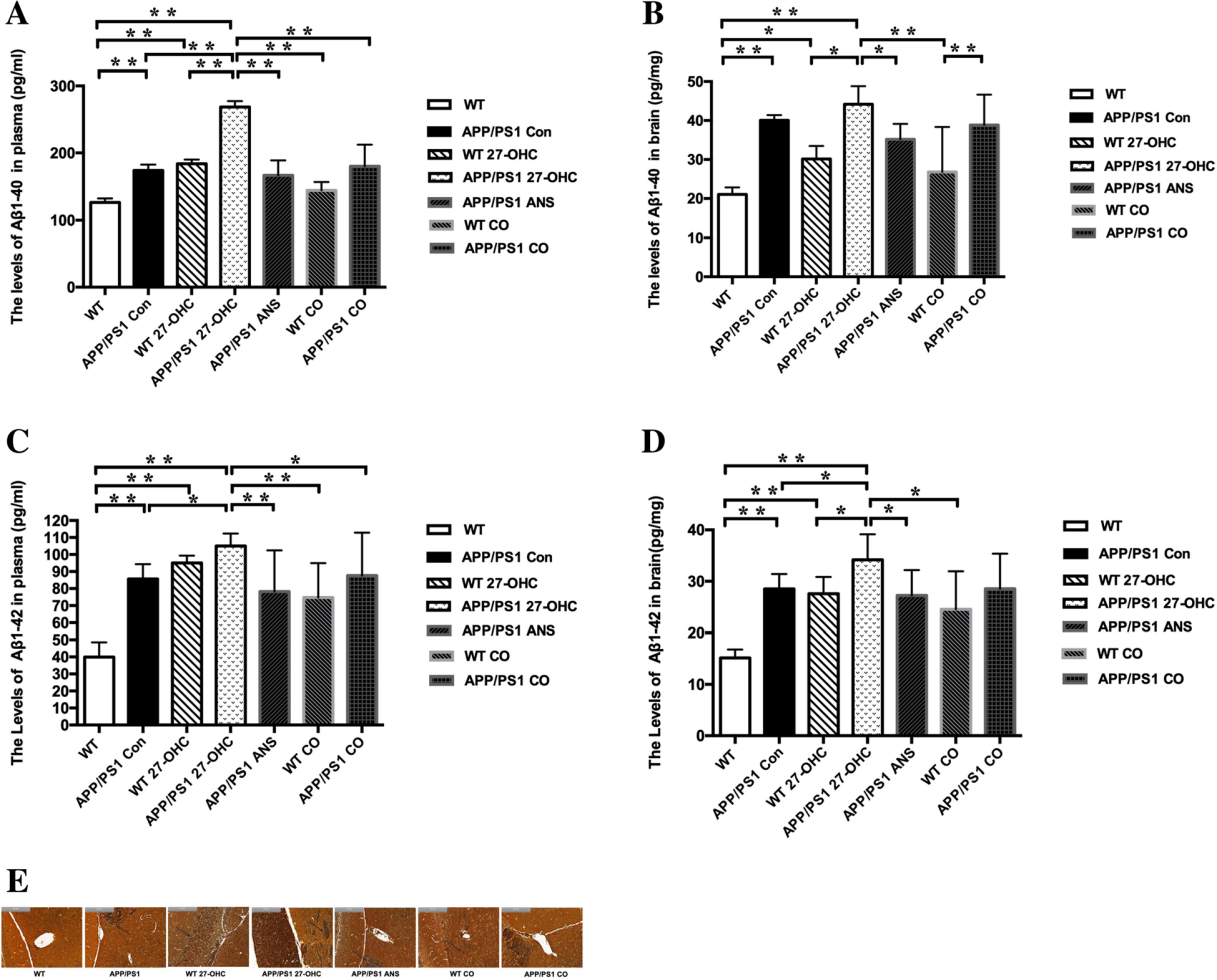
The plasma and brain levels of Aβ1-40 (**a**, **b**) and Aβ1-42 (**c**, **d**) and silver staining of Aβ plaques (**e**) of each group. Groups: WT: wild-type control group of C57BL/6J mice; APP/PS1 Con: transgenic control group of APP/PS1 mice; WT 27-OHC: C57BL/6J mice treated with 5.5 mg/kg 27-hydroxycholesterol; APP/PS1 27-OHC: APP/PS1 mice treated with 5.5 mg/kg 27-hydroxycholesterol; APP/PS1 ANS: APP/PS1 mice treated with 0.2 mg/day anastrozole; WT CO: C57BL/6J mice treated with 5.5 mg/kg 27-hydroxycholesterol plus 0.2 mg/day anastrozole; APP/PS1 CO: APP/PS1 mice treated with 5.5 mg/kg 27-hydroxycholesterol plus 0.2 mg/day anastrozole. One-way analysis of variance (ANOVA) was performed and post hoc comparisons were carried out using the LSD test. Asterisks indicate significant differences. Data are presented as mean ± SEM. *n* = 10/group. *P < 0.05; **P < 0.01

In addition, the plasma and brain loads of Aβ1-40 and Aβ1-42 were significantly aggravated in 27-OHC-treated APP/PS1 mice compared with the APP/PS1 control group, while ANS and 27-OHC plus ANS treatment significantly reversed these changes (Fig. 8a–d).

Simultaneously, Bielschowsky silver staining of Aβ in brain sections also showed that 27-OHC-treated APP/PS1 mice exhibited marked Aβ plaques deposition (Fig. 8e). What is more, APP/PS1 control mice exhibited significantly increased intestine and brain levels of 27-OHC, liver TC, TG, and LDL-C as well as brain and plasma levels of Aβ load than WT mice, suggesting pre-existing neuropathological changes in APP/PS1 mice.

### 27-OHC treatment aggravated cognitive deficits

**Fig. 9:**
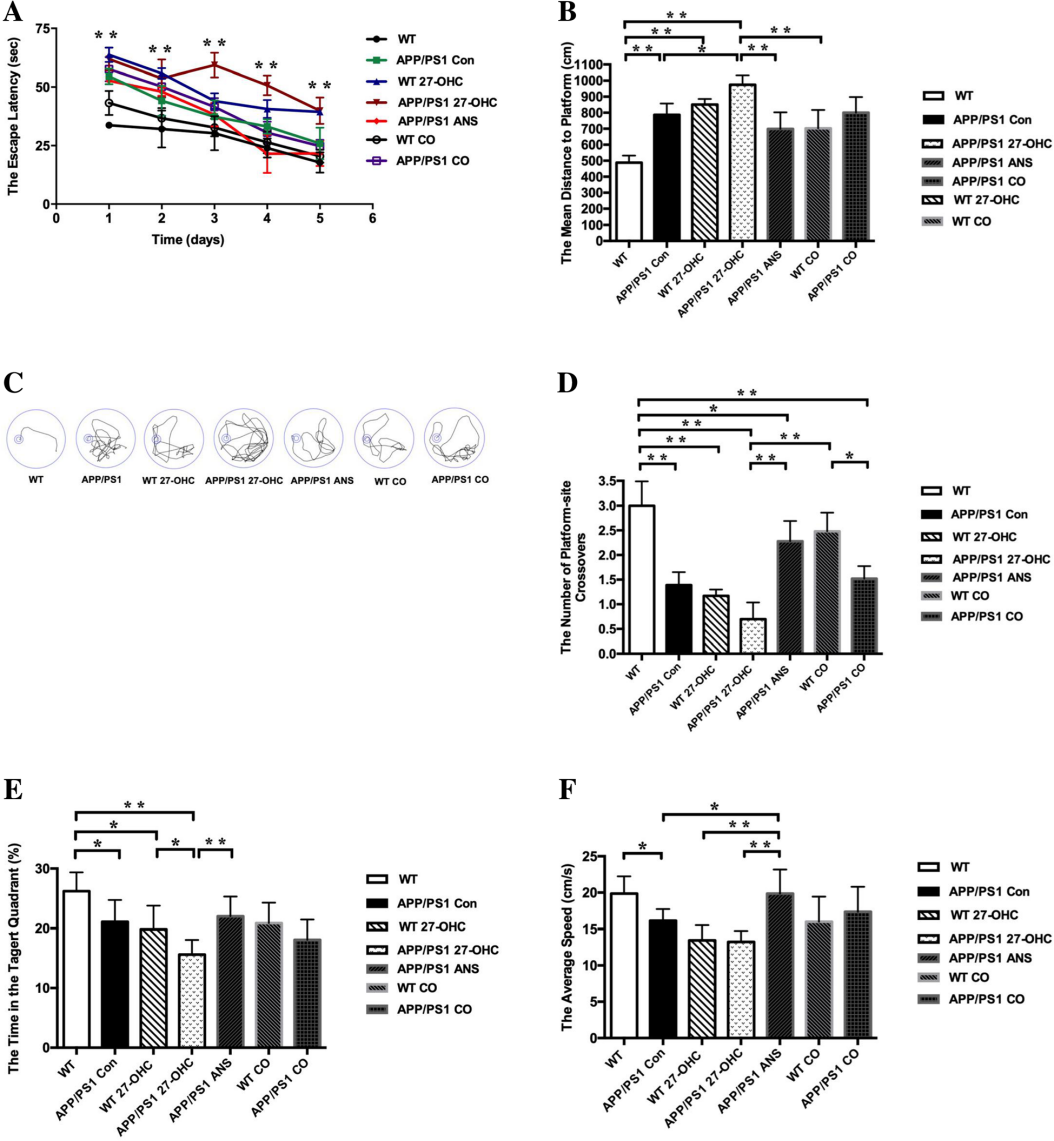
The escape latency (**a**), escape distance (**b**), and representative images of path (**c**) in orientation navigation test and the crossing-target number (**d**), the target-quadrant abidance (**e**), and swimming speed (**f**) and in spatial probe test determined with the Morris water maze in different groups. Groups: WT: wild-type control group of C57BL/6J mice; APP/PS1 Con: transgenic control group of APP/PS1 mice; WT 27-OHC: C57BL/6J mice treated with 5.5 mg/kg 27-hydroxycholesterol; APP/PS1 27-OHC: APP/PS1 mice treated with 5.5 mg/kg 27-hydroxycholesterol; APP/PS1 ANS: APP/PS1 mice treated with 0.2 mg/day anastrozole; WT CO: C57BL/6J mice treated with 5.5 mg/kg 27-hydroxycholesterol plus 0.2 mg/day anastrozole; APP/PS1 CO: APP/PS1 mice treated with 5.5 mg/kg 27-hydroxycholesterol plus 0.2 mg/day anastrozole. One-way analysis of variance (ANOVA) was performed and post hoc comparisons were carried out using the LSD test. Asterisks indicate significant differences. Data are presented as mean ± SEM. *n* = 10/group. *P < 0.05; **P < 0.01

To assess the spatial learning and memory ability of the mice, the Morris water maze test was performed including orientation navigation and spatial probe tests. The escape latency to the target platform during the 5 days of training for all groups is shown in Fig. 9a. As a well-established mouse model of AD, APP/PS1 mice exhibited significantly higher latency to locate the platform in the Morris water maze test than WT C57BL/6J mice because of the overproduction of Aβ peptide in brains of these animals.

We found that 27-OHC administration to APP/PS1 and WT mice significantly increased their latency to locate the platform (*P* < 0.05). Interestingly, the ANS-treated APP/PS1 group preferentially performed a selective search for the platform and had a shorter distance to the platform than the 27-OHC-treated APP/PS1 group (Fig. 9b). In addition, no significant difference was detected between ANS-treated APP/PS1 mice and 27-OHC + ANS-treated APP/PS1 mice (*P* > 0.05).

These findings were supported by representative images of the path of travel during the orientation navigation, which demonstrated a longer path length of APP/PS1 or WT mice treated with 27-OHC than the APP/PS1 or WT control mice, or the ANS alone treated mice (Fig. 9c).

In the spatial probe test, compared to the WT mice, the APP/PS1 group mice swam more randomly throughout the tank, suggesting poor memory retention of the location of the platform. The 27-OHC-treated APP/PS1 or WT group showed an obvious decrease in the number of target crossings (Fig. 9d) and target-quadrant abidance (Fig. 9e) compared to the control groups (*P* < 0.05), while the ANS-treated APP/PS1 group completely reversed this trend. 27-OHC + ANS-treated mice showed no significant differences in the number of target crossings or the target-quadrant abidance in comparison to the control mice (*P* > 0.05). For swimming speed, the ANS-treated APP/PS1 group had a significantly increased speed relative to both the control groups and the 27-OHC-treated groups (Fig. 9f, *P* < 0.05).

**Fig. 10:**
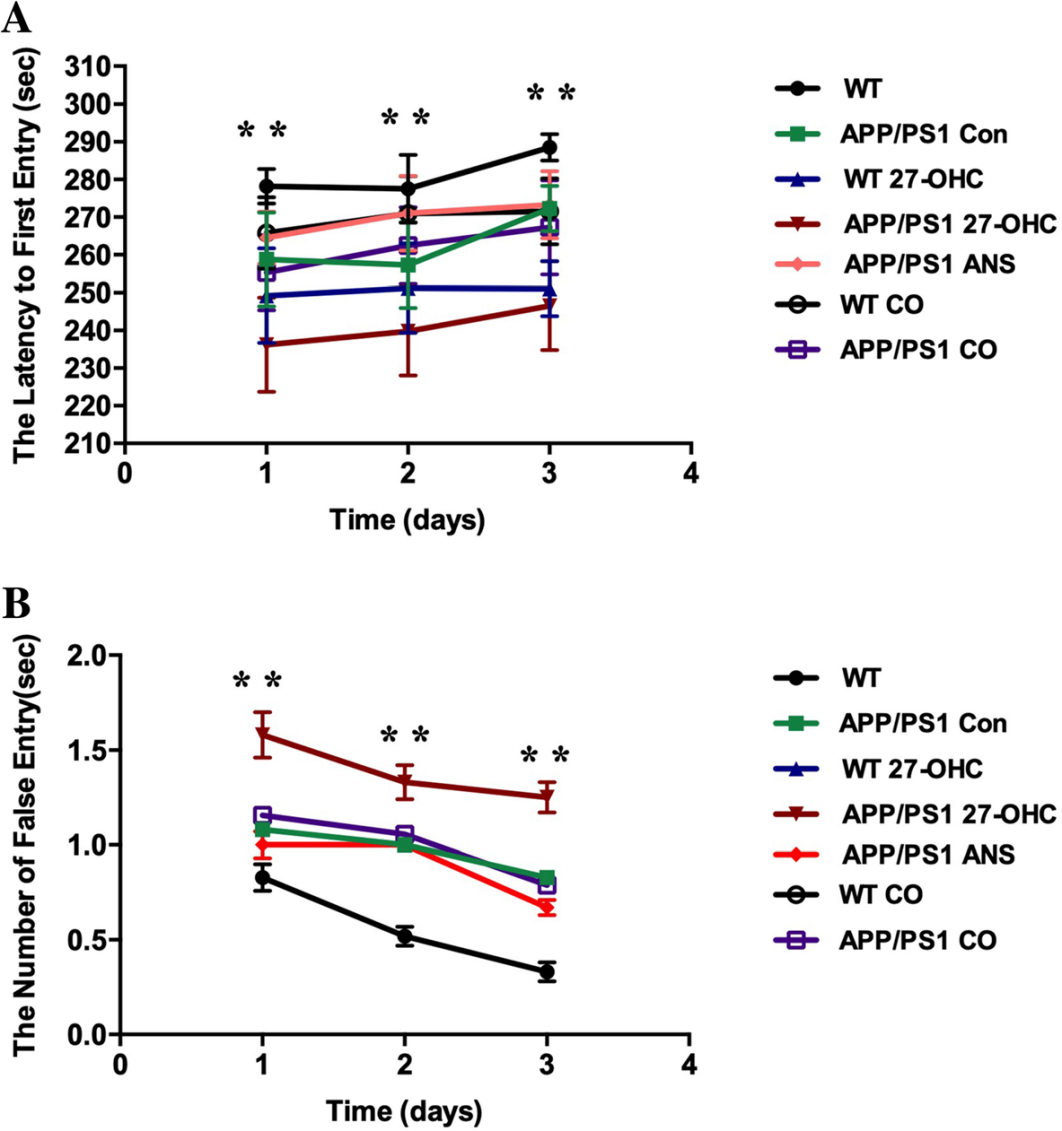
The latency to enter the dark area (**a**) and the frequency of entries to the dark area (**b**) determined with the passive avoidance test in different groups. Groups: WT: wild-type control group of C57BL/6J mice; APP/PS1 Con: transgenic control group of APP/PS1 mice; WT 27-OHC: C57BL/6J mice treated with 5.5 mg/kg 27-hydroxycholesterol; APP/PS1 27-OHC: APP/PS1 mice treated with 5.5 mg/kg 27-hydroxycholesterol; APP/PS1 ANS: APP/PS1 mice treated with 0.2 mg/day anastrozole; WT CO: C57BL/6J mice treated with 5.5 mg/kg 27-hydroxycholesterol plus 0.2 mg/day anastrozole; APP/PS1 CO: APP/PS1 mice treated with 5.5 mg/kg 27-hydroxycholesterol plus 0.2 mg/day anastrozole. One-way analysis of variance (ANOVA) was performed and post hoc comparisons were carried out using the LSD test. Asterisks indicate significant differences. Data are presented as mean ± SEM. *n* = 10/group. *P < 0.05; **P < 0.01

In the passive avoidance test, compared to the APP/PS1 and WT control groups, the latency to enter the dark area was significantly shortened and the frequency of entries into the dark compartment was increased in the 27-OHC-treated group (Fig. 10a, b, *P*< 0.05), suggesting that 27-OHC treatment could induce learning and memory impairment. Both the ANS alone-treated and 27-OHC + ANS-treated groups had significantly prolonged latencies of entry into the dark area and had decreased frequencies of entries into the dark compartment (*P* < 0.05). These results indicated that the high dose of 27-OHC could result in spatial learning and memory capability impairment while inhibition of 27-OHC synthesis could prevent or reverse the cognitive deficits induced by 27-OHC in the APP/PS1 double transgenic mice.

### 27-OHC treatment disrupted the microbial composition and affected SCFA levels

**Fig. 11:**
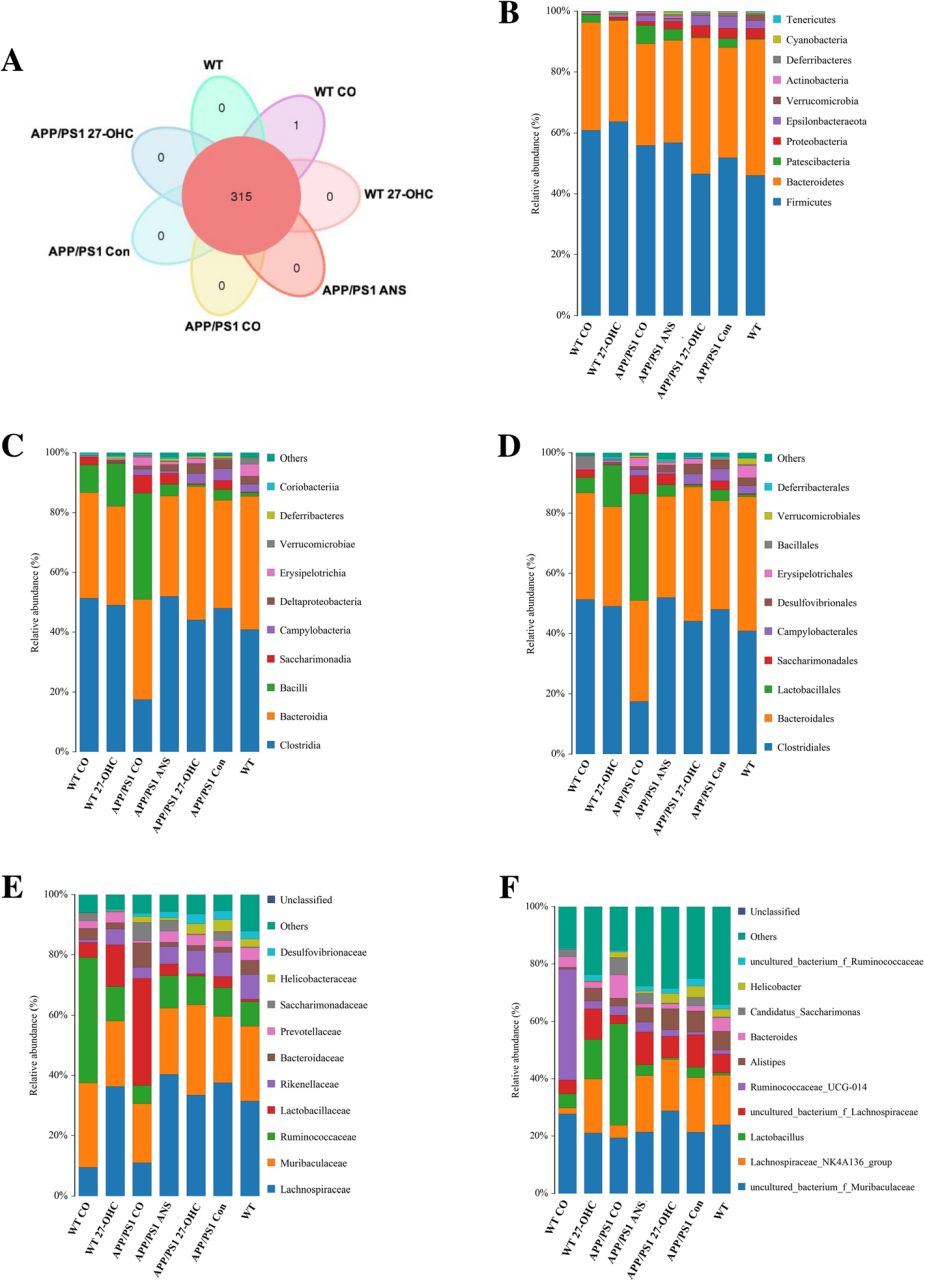
**a** Venn diagram illustrated the overlap of the OTUs identified in fecal microbiota among the five groups. Relative abundance of phylum level (**b**), class level (**c**), order level (**d**), family level (**e**), and genera level (**f**) gut microbial taxa. Groups: WT: wild-type control group of C57BL/6J mice; APP/PS1 Con: transgenic control group of APP/PS1 mice; WT 27-OHC: C57BL/6J mice treated with 5.5 mg/kg 27-hydroxycholesterol; APP/PS1 27-OHC: APP/PS1 mice treated with 5.5 mg/kg 27-hydroxycholesterol; APP/PS1 ANS: APP/PS1 mice treated with 0.2 mg/day anastrozole; WT CO: C57BL/6J mice treated with 5.5 mg/kg 27-hydroxycholesterol plus 0.2 mg/day anastrozole; APP/PS1 CO: APP/PS1 mice treated with 5.5 mg/kg 27-hydroxycholesterol plus 0.2 mg/day anastrozole. *n* = 6/group

To assess the influence of 27-OHC treatment on the gut microbiota in mice, sequencing of the V3–V4 regions of the 16S rDNA genes was conducted on fecal samples. As shown in the Venn diagram, a total of 315 operational taxonomic units (OTUs) were displayed at a 97% similarity level and shared among all samples (Fig. 11a). The overall microbial compositions were evaluated at different taxonomic levels (Fig. 11b–f). Cotreatment with 27-OHC and ANS vigorously changed the microbiome and the profile of the microbial composition of the 27-OHC-treated WT mice clustered more closely to the APP/PS1 control mice than to the profile of the WT control mice.

**Fig. 12:**
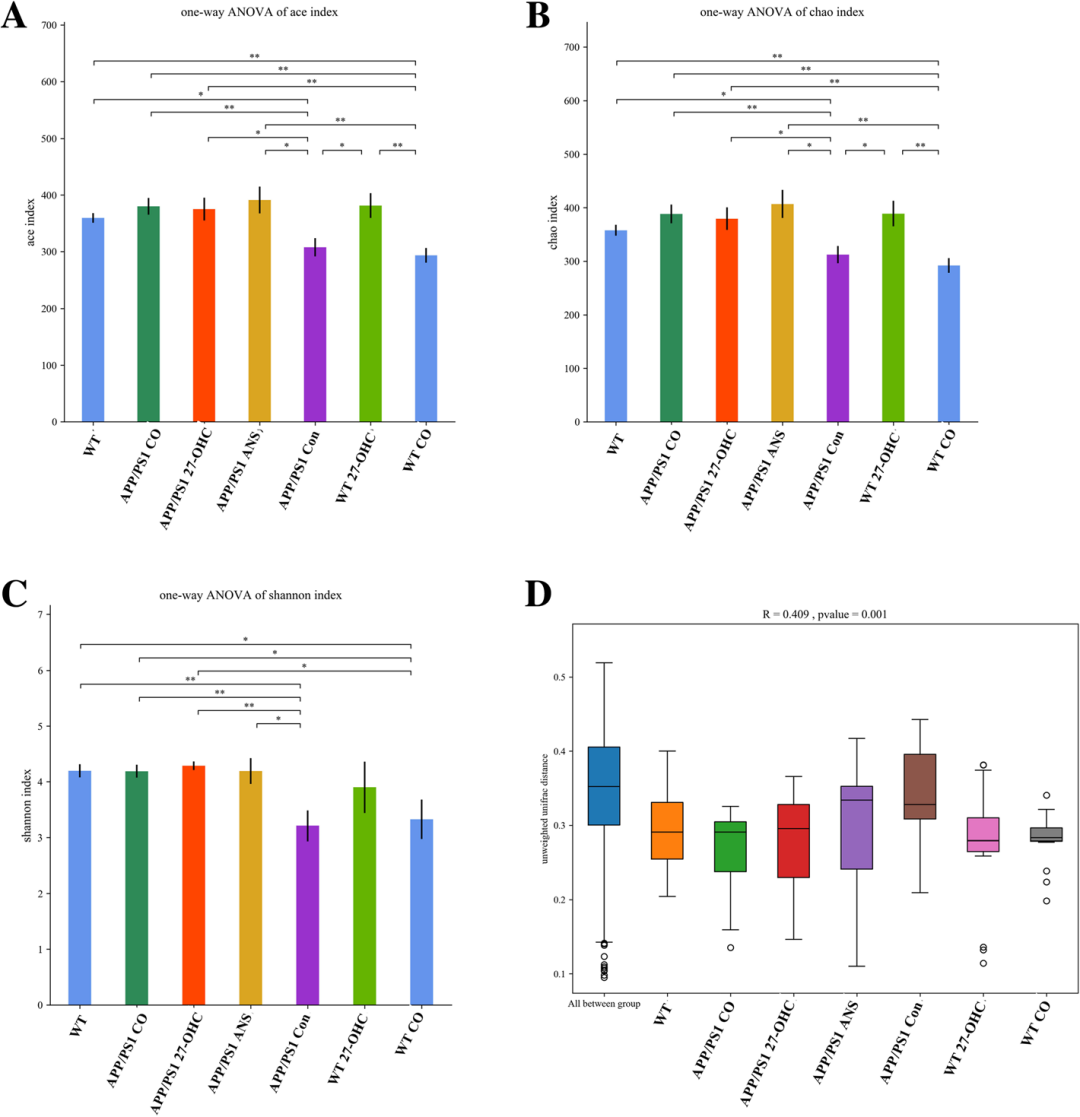
The α-diversity of the fecal microbiome among seven groups according to Ace (**a**), Chao 1 (**b**), and Shannon index (**c**). Data are presented as mean ± SD. One-way analysis of variance (ANOVA) was performed and post hoc comparisons were carried out using the LSD test. Asterisks indicate significant differences. *P < 0.05; **P < 0.01. The β-diversity of the fecal microbiome among five groups according to unweighted UniFrac distance (**d**). Each box plot represents the median, interquartile range, minimum, and maximum values. Groups: WT: wild type control group of C57BL/6J mice; APP/PS1 Con: transgenic control group of APP/PS1 mice; WT 27-OHC: C57BL/6J mice treated with 5.5 mg/kg 27-hydroxycholesterol; APP/PS1 27-OHC: APP/PS1 mice treated with 5.5 mg/kg 27-hydroxycholesterol; APP/PS1 ANS: APP/PS1 mice treated with 0.2 mg/day anastrozole; WT CO: C57BL/6J mice treated with 5.5 mg/kg 27-hydroxycholesterol plus 0.2 mg/day anastrozole; APP/PS1 CO: APP/PS1 mice treated with 5.5 mg/kg 27-hydroxycholesterol plus 0.2 mg/day anastrozole. *n* = 6/group

Fecal microbiota diversity within a single sample, namely α diversity, was measured using three indices, the Ace, Chao1, and Shannon indices. As shown in Fig. 12a–c, cotreatment with 27-OHC and ANS of APP/PS1 mice resulted in a significantly lower commensal diversity than in the ANS-treated group while cotreatment of WT mice resulted in a lower diversity than in the 27-OHC-treated group (*P* < 0.05). To assess the overall structure of the gut microbiota, β diversity analysis for interindividual microbiota relatedness using unweighted UniFrac distance demonstrated that the structure and composition of the microbiota differed significantly among groups (*P* = 0.001, Fig. 12d).

**Fig. 13:**
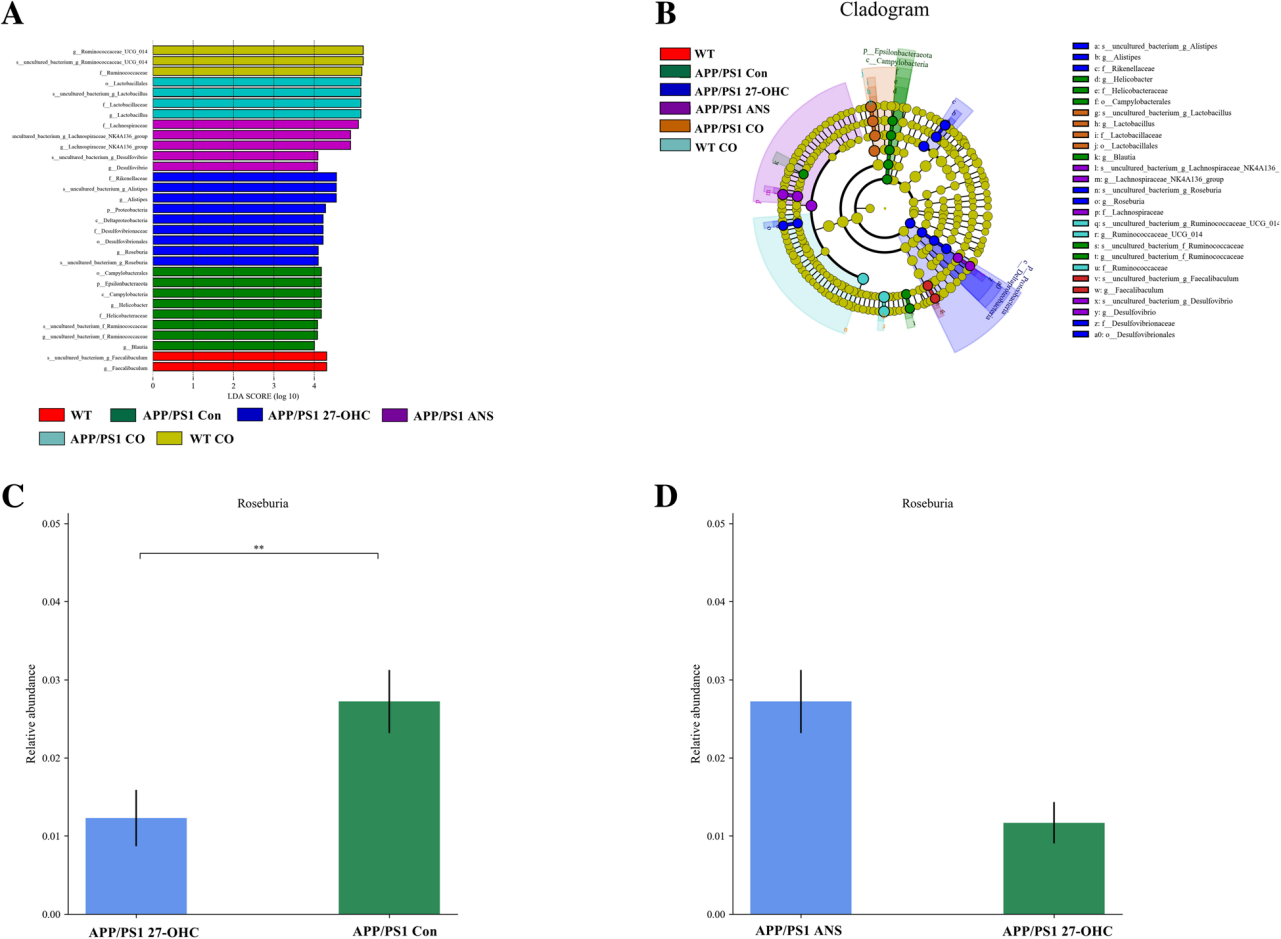
Comparison of the representative taxonomic abundance among different groups. **a** Linear discriminant analysis (LDA) effect size (LEfSe) analysis revealed significant bacterial differences in fecal microbiota between different groups. The LDA scores (log10) > 4 and *P* < 0.05 are listed. **b** Cladogram using LEfSe method indicating the phylogenetic distribution of fecal microbiota associated with different groups. Metastats analysis indicated the significant differences in *Roseburia* between two groups of APP/PS1 group and APP/PS1 27-OHC group (**c**). Metastats analysis indicated the significant differences in *Roseburia* between two groups of APP/PS1 27-OHC group and APP/PS1 ANS group (**b**). *p* phylum, *c* class, *o* order, *f* family, *g* genus. Groups: WT: wild-type control group of C57BL/6J mice; APP/PS1 Con: transgenic control group of APP/PS1 mice; WT 27-OHC: C57BL/6J mice treated with 5.5 mg/kg 27-hydroxycholesterol; APP/PS1 27-OHC: APP/PS1 mice treated with 5.5 mg/kg 27-hydroxycholesterol; APP/PS1 ANS: APP/PS1 mice treated with 0.2 mg/day anastrozole; WT CO: C57BL/6J mice treated with 5.5 mg/kg 27-hydroxycholesterol plus 0.2 mg/day anastrozole; APP/PS1 CO: APP/PS1 mice treated with 5.5 mg/kg 27-hydroxycholesterol plus 0.2 mg/day anastrozole. *n* = 6/group

A supervised comparison of the microbiota among different groups was conducted by utilizing the linear discriminant analysis (LDA) effect size (LEfSe). We used a logarithmic LDA score cutoff of 4.0 to identify important taxonomic differences among the five groups and the representative cladogram showed variant taxa at different taxonomic levels (Fig. 13a, b). Here, we particularly found differences in the taxa at the genus level. Metastats analysis showed that the relative abundance of the genera *Roseburia* was significantly lower in the 27-OHC-treated APP/PS1 mice than in the APP/PS1 control mice and the ANS-treated APP/PS1 mice (Fig. 13c, d). As a butyrate producer, *Roseburia* can metabolize dietary fibers and exhibit anti-inflammatory effects in the gut.

**Fig. 14:**
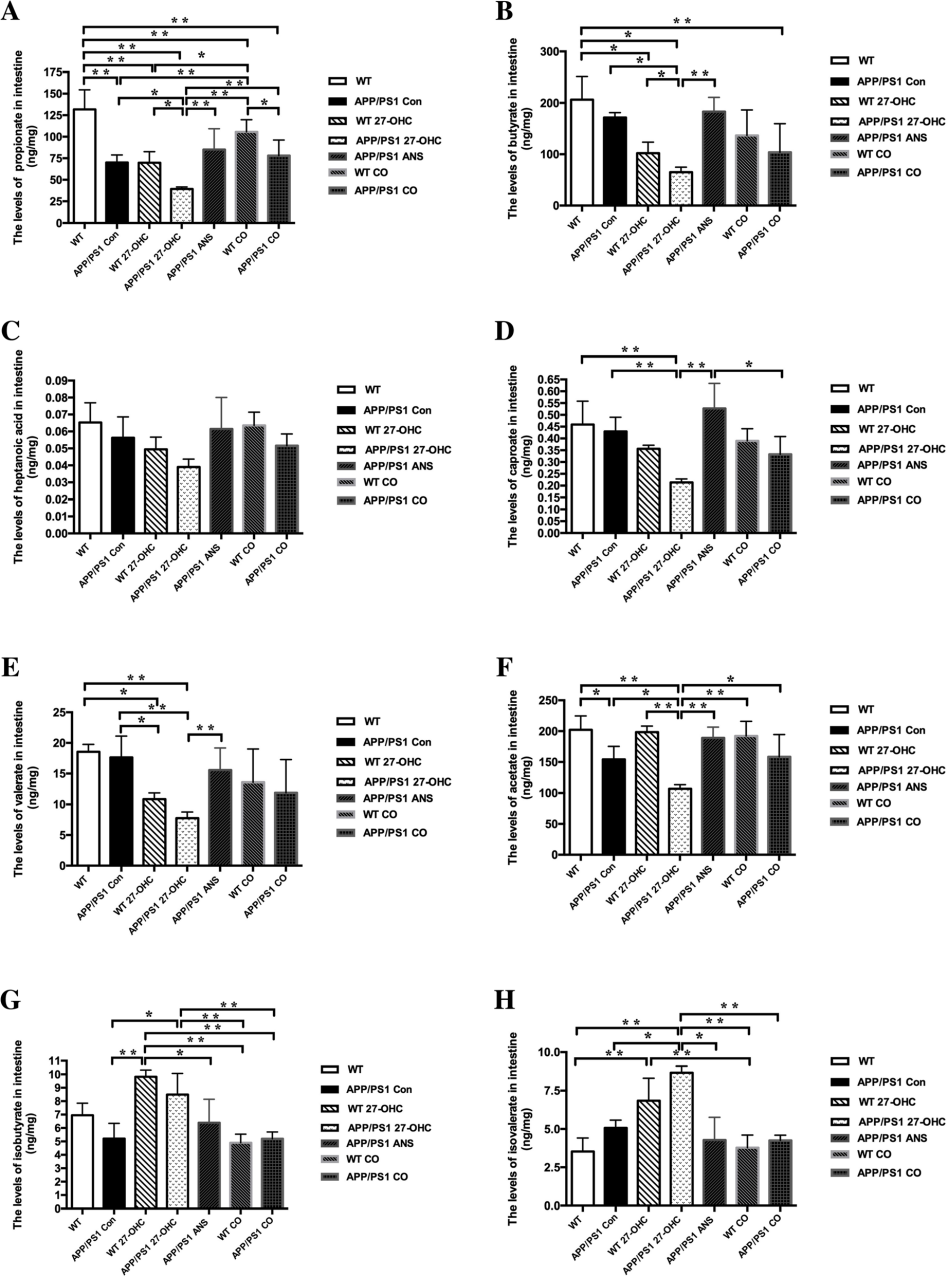
The fecal levels of propionate (**a**), butyrate (**b**), heptanoic acid (**c**), caproate (**d**), valerate (**e**), acetate (**f**), isobutyrate (**g**), and isovalerate (**h**) in different groups. Groups: WT: wild-type control group of C57BL/6J mice; APP/PS1 Con: transgenic control group of APP/PS1 mice; WT 27-OHC: C57BL/6J mice treated with 5.5 mg/kg 27-hydroxycholesterol; APP/PS1 27-OHC: APP/PS1 mice treated with 5.5 mg/kg 27-hydroxycholesterol; APP/PS1 ANS: APP/PS1 mice treated with 0.2 mg/day anastrozole; WT CO: C57BL/6J mice treated with 5.5 mg/kg 27-hydroxycholesterol plus 0.2 mg/day anastrozole; APP/PS1 CO: APP/PS1 mice treated with 5.5 mg/kg 27-hydroxycholesterol plus 0.2 mg/day anastrozole. One-way analysis of variance (ANOVA) was performed and post hoc comparisons were carried out using the LSD test. Asterisks indicate significant differences. Data are presented as mean ± SEM. *P < 0.05; **P < 0.01. *n* = 6/group

We also explored alterations of fecal SCFA profiles caused by 27-OHC (Fig. 14a–h). Our results indicated that fecal levels of propionate, butyrate, caproate, valerate, and acetate were significantly lower in the 27-OHC-treated mice than in the control and ANS-treated mice (*P* < 0.05). However, the isobutyrate and isovalerate levels were significantly higher in the 27-OHC-treated mice than in the control and ANS-treated mice (*P* < 0.05). There were no significant differences in heptanoic acid among the groups (*P* > 0.05).

### 27-OHC treatment induced intestinal pathology

**Fig. 15:**
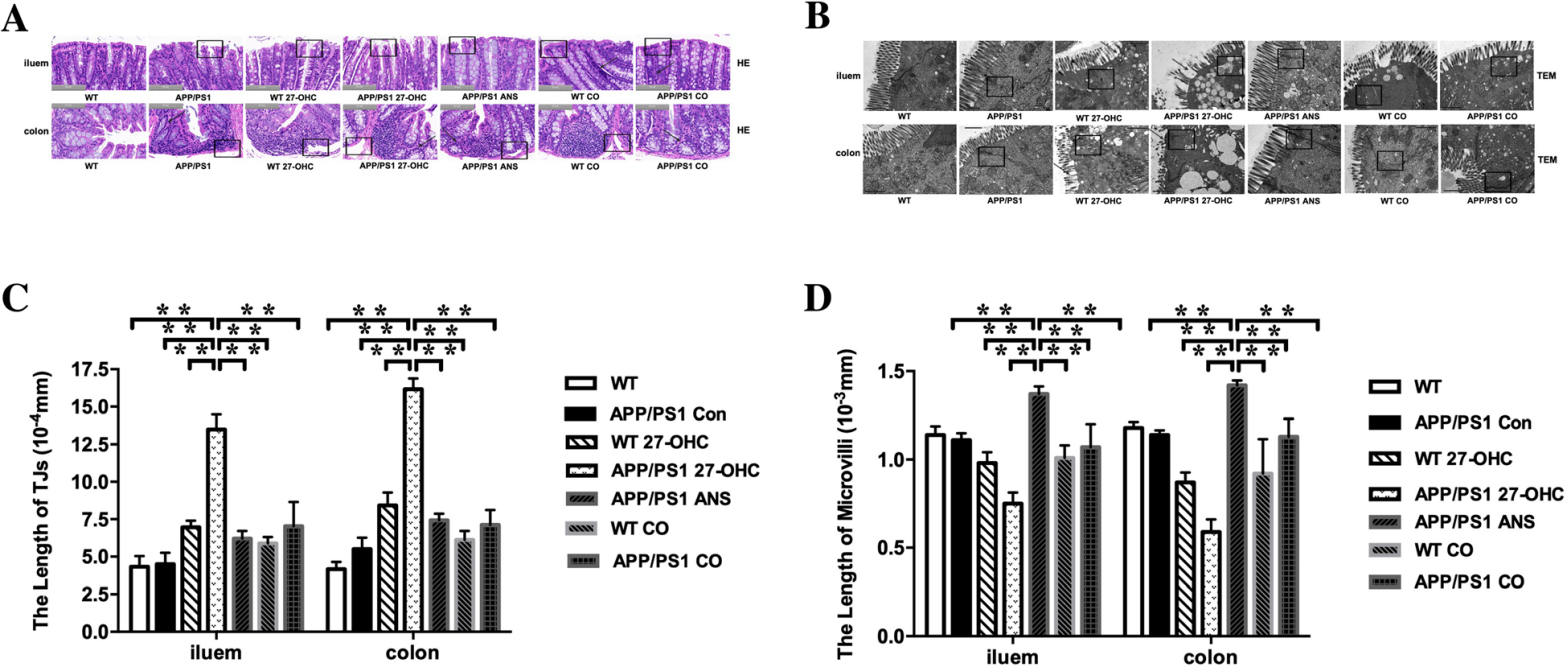
H&E staining was performed to assess the morphology of the ileum and colon collected from different groups (**a**). Transmission electron micrographs of ultrastructure in ileum and colon (**b**). *n* = 6/group. Quantitative analyses revealed intercellular ultrastructure indicated by length of tight junctions (TJs, **c**) and microvilli (**d**). *TJs* tight junctions. Groups: WT: wild-type control group of C57BL/6J mice; APP/PS1 Con: transgenic control group of APP/PS1 mice; WT 27-OHC: C57BL/6J mice treated with 5.5 mg/kg 27-hydroxycholesterol; APP/PS1 27-OHC: APP/PS1 mice treated with 5.5 mg/kg 27-hydroxycholesterol; APP/PS1 ANS: APP/PS1 mice treated with 0.2 mg/day anastrozole; WT CO: C57BL/6J mice treated with 5.5 mg/kg 27-hydroxycholesterol plus 0.2 mg/day anastrozole; APP/PS1 CO: APP/PS1 mice treated with 5.5 mg/kg 27-hydroxycholesterol plus 0.2 mg/day anastrozole. One-way analysis of variance (ANOVA) was performed and post hoc comparisons were carried out using the LSD test. Asterisks indicate significant differences. Data are presented as mean ± SEM. *P < 0.05; **P < 0.01. *n* = 6/group

To investigate the impact of 27-OHC on the intestinal pathology, histopathological changes of the ileum and colon were examined. As shown in Fig. 15a, necrotic and ulcerative changes together with mucosal atrophy and destruction of the villi were noted in the ileum of 27-OHC-treated mice, whereas the villi of the APP/PS1 and WT control mice were intact and there was no epithelial disruption. However, ANS treatment resulted in significant attenuation of the ileal lesions in the mice. Necrotic and ulcerative lesions were also observed in the colon in 27-OHC-treated mice compared to the approximately intact histology observed in the APP/PS1 and WT control mice. ANS treatment, however, alleviated the pathological changes in the colon.

Next, we applied transmission electron microscopy for ultrastructural analysis of tight junctions and microvilli in the ileum and colon. Figure 15b shows selected micrographs of the ultrastructural analysis of each experimental group. Qualitative and quantitative analyses revealed that APP/PS1 and WT control mice showed a relatively intact apical intercellular ultrastructure, whereas 27-OHC-treated mice were morphologically indistinguishable from controls, as indicated by dilated (larger) intercellular spaces in the tight junctions (Fig. 15c) and shorter intestine microvilli with less density (Fig. 15d). However, the tight junctions and microvilli of the ANS-treated and 27-OHC + ANS-treated groups appeared to be better preserved, which suggests that the tight junctions and microvilli are maintained to some extent in response to ANS.

### 27-OHC treatment increased intestinal barrier permeability

**Fig. 16:**
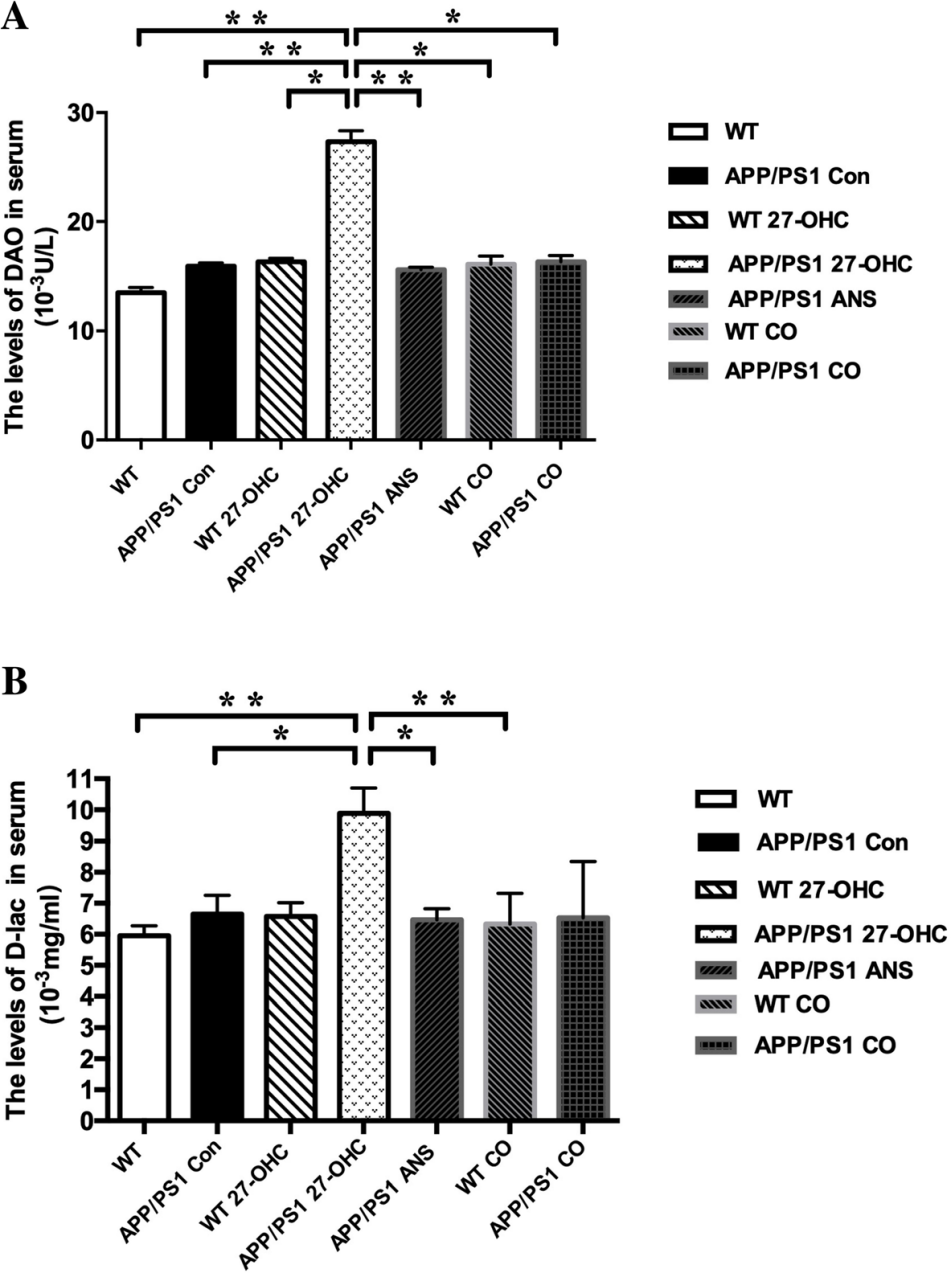
Comparisons of serum levels DAO (**a**) and d-lactate (**b**) for different groups. Groups: WT: wild-type control group of C57BL/6J mice; APP/PS1 Con: transgenic control group of APP/PS1 mice; WT 27-OHC: C57BL/6J mice treated with 5.5 mg/kg 27-hydroxycholesterol; APP/PS1 27-OHC: APP/PS1 mice treated with 5.5 mg/kg 27-hydroxycholesterol; APP/PS1 ANS: APP/PS1 mice treated with 0.2 mg/day anastrozole; WT CO: C57BL/6J mice treated with 5.5 mg/kg 27-hydroxycholesterol plus 0.2 mg/day anastrozole; APP/PS1 CO: APP/PS1 mice treated with 5.5 mg/kg 27-hydroxycholesterol plus 0.2 mg/day anastrozole. One-way analysis of variance (ANOVA) was performed and post hoc comparisons were carried out using the LSD test. Asterisks indicate significant differences. Data are presented as mean ± SEM. *P < 0.05; **P < 0.01. *n* = 6/group

To evaluate the severity of intestinal barrier dysfunction, serum DAO and d-lactate were measured as indicators of intestinal permeability. As shown in Fig. 16a, b, compared with the APP/PS1 and WT control groups, serum DAO and d-lactate were both significantly increased in the 27-OHC-treated APP/PS1 group (all *P* < 0.05). The ANS and 27-OHC + ANS groups had significantly lower levels of serum DAO and d-lactate than those of the control groups (*P* < 0.05, respectively).

**Fig. 17:**
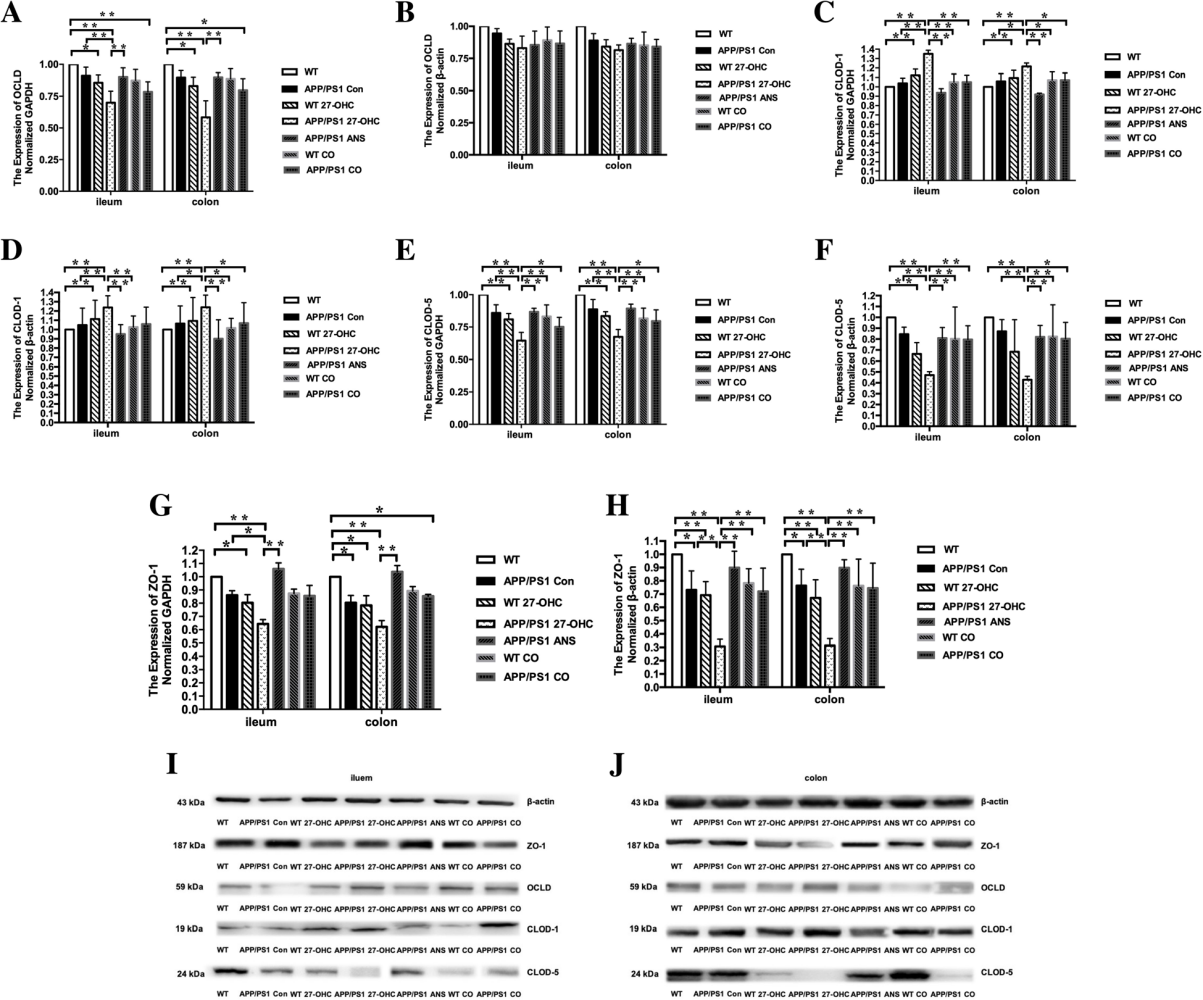
Relative mRNA levels (**a**) and Western blot analyses (**b**) of OLCD, relative mRNA levels (**c**) and Western blot analyses (**d**) of CLOD-1, relative mRNA levels (**e**) and Western blot analyses (**f**) of CLOD-5, and relative mRNA levels (**g**) and Western blot analyses (**h**) of ZO-1 expression for different groups. Protein bands for Western blot analyses of tight junction protein expression for different groups in iluem (I) and colon (**j**). Groups: WT: wild-type control group of C57BL/6J mice; APP/PS1 Con: transgenic control group of APP/PS1 mice; WT 27-OHC: C57BL/6J mice treated with 5.5 mg/kg 27-hydroxycholesterol; APP/PS1 27-OHC: APP/PS1 mice treated with 5.5 mg/kg 27-hydroxycholesterol; APP/PS1 ANS: APP/PS1 mice treated with 0.2 mg/day anastrozole; WT CO: C57BL/6J mice treated with 5.5 mg/kg 27-hydroxycholesterol plus 0.2 mg/day anastrozole; APP/PS1 CO: APP/PS1 mice treated with 5.5 mg/kg 27-hydroxycholesterol plus 0.2 mg/day anastrozole. One-way analysis of variance (ANOVA) was performed and post hoc comparisons were carried out using the LSD test. Asterisks indicate significant differences. Data are presented as mean ± SEM. *P < 0.05; **P < 0.01. *n* = 6/group

The ileal and colonic gene and protein expression of tight junction proteins was further analyzed to confirm the detrimental effects of 27-OHC treatment on the intestinal integrity. As shown in Fig. 17, compared to the APP/PS 1 control and WT control mice, the ileal (Fig. 17i) and colonic (Fig. 17j) gene and protein expression levels of occludin (Fig. 17a, b), claudin 1 (Fig. 17c, d), claudin 5 (Fig. 17e, f), and ZO-1 (Fig. 17g, h) were significantly downregulated in 27-OHC-treated APP/PS1 mice (*P* < 0.05), except that no significant changes were observed in the protein expression levels of occludin (*P* > 0.05). In contrast, the ileal and colonic expression was markedly increased as a result of ANS or 27-OHC plus ANS treatment (*P* < 0.05).

**Fig. 18:**
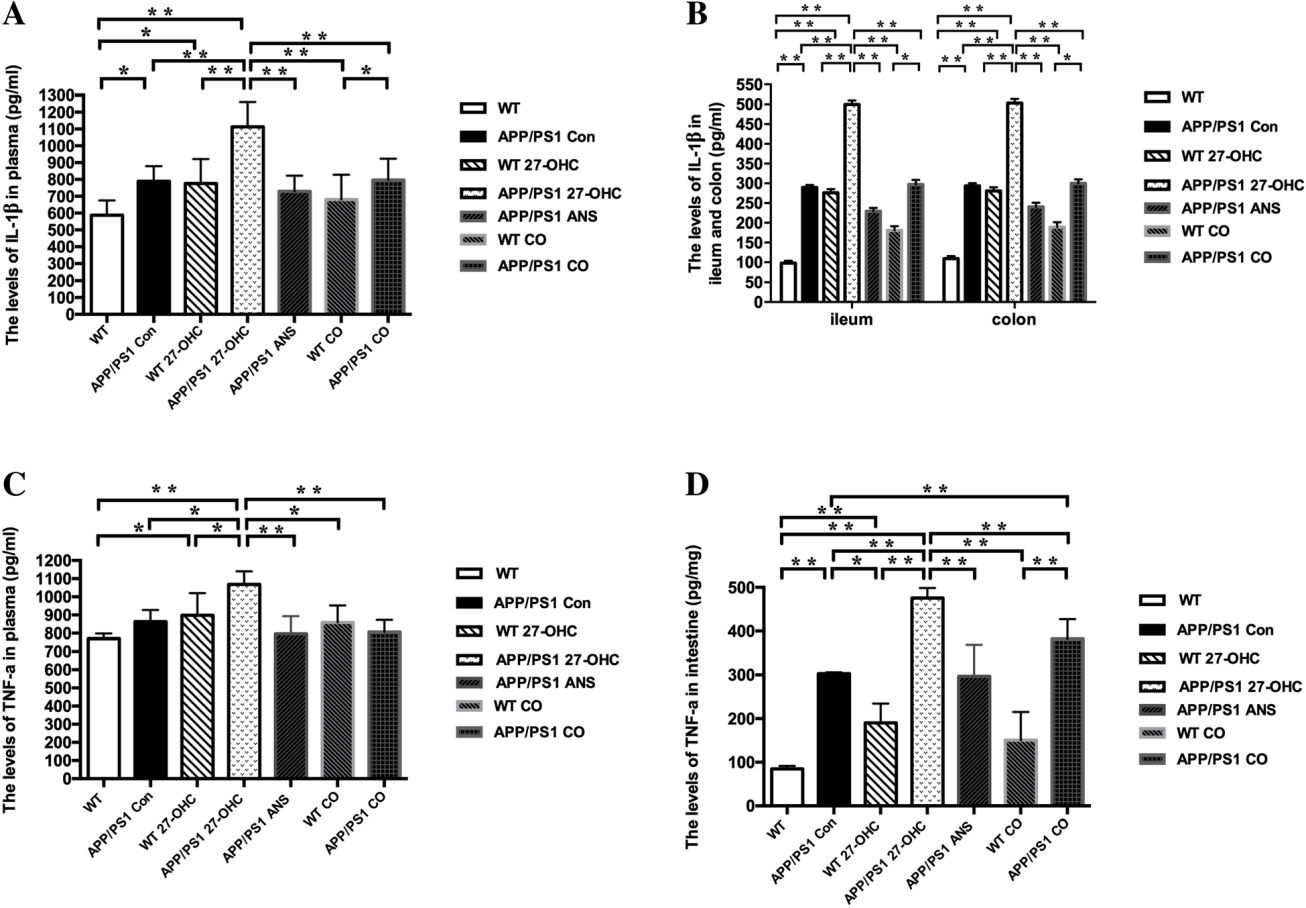
The levels of IL-1β in plasma (**a**) and ileum and colon (**b**), TNF-α in plasma (**c**) and ileum and colon (**d**) for different groups. Groups: WT: wild-type control group of C57BL/6J mice; APP/PS1 Con: transgenic control group of APP/PS1 mice; WT 27-OHC: C57BL/6J mice treated with 5.5 mg/kg 27-hydroxycholesterol; APP/PS1 27-OHC: APP/PS1 mice treated with 5.5 mg/kg 27-hydroxycholesterol; APP/PS1 ANS: APP/PS1 mice treated with 0.2 mg/day anastrozole; WT CO: C57BL/6J mice treated with 5.5 mg/kg 27-hydroxycholesterol plus 0.2 mg/day anastrozole; APP/PS1 CO: APP/PS1 mice treated with 5.5 mg/kg 27-hydroxycholesterol plus 0.2 mg/day anastrozole. One-way analysis of variance (ANOVA) was performed and post hoc comparisons were carried out using the LSD test. Asterisks indicate significant differences. Data are presented as mean ± SEM. *P < 0.05; **P < 0.01. *n* = 6/group

**Fig. 19:**
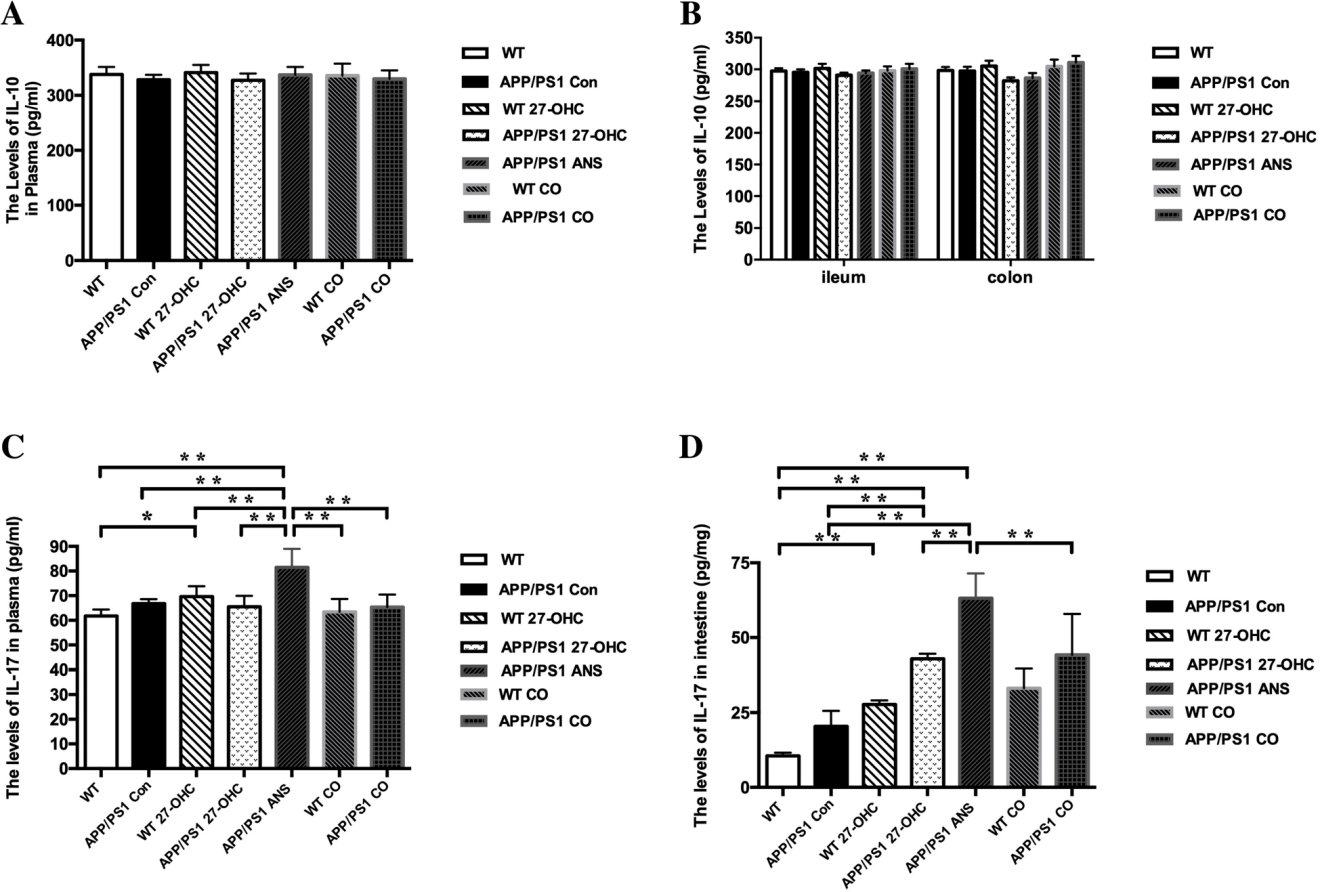
The levels of IL-10 in plasma (**a**) and ileum and colon (**b**) and IL-17 in plasma (**c**) and ileum and colon (**d**) for different groups. Groups: WT: wild-type control group of C57BL/6J mice; APP/PS1 Con: transgenic control group of APP/PS1 mice; WT 27-OHC: C57BL/6J mice treated with 5.5 mg/kg 27-hydroxycholesterol; APP/PS1 27-OHC: APP/PS1 mice treated with 5.5 mg/kg 27-hydroxycholesterol; APP/PS1 ANS: APP/PS1 mice treated with 0.2 mg/d anastrozole; WT CO: C57BL/6J mice treated with 5.5 mg/kg 27-hydroxycholesterol plus 0.2 mg/day anastrozole; APP/PS1 CO: APP/PS1 mice treated with 5.5 mg/kg 27-hydroxycholesterol plus 0.2 mg/day anastrozole. One-way analysis of variance (ANOVA) was performed and post hoc comparisons were carried out using the LSD test. Asterisks indicate significant differences. Data are presented as mean ± SEM. *P < 0.05, **P < 0.01. *n* = 6/group

We evaluated systemic and intestinal inflammation by measuring the levels of the pro-inflammatory cytokines IL-1β and TNF-α as well as the levels of the anti-inflammatory cytokines IL-10 and IL-17 in the plasma and intestinal tissue. As shown in Fig. 18a–d, there were significantly increased levels of IL-1β in the plasma, ileum, and colon in the 27-OHC-treated WT and APP/PS1 groups (*P* < 0.05) compared with the WT and APP/PS1 control groups. The ANS- and 27-OHC + ANS-treated APP/PS1 groups had significantly lower levels of IL-1β than the 27-OHC-treated APP/PS1 group (*P* < 0.05). Moreover, the APP/PS1 mice also had significantly higher IL-1β levels in the plasma, ileum, and colon than the WT mice. In regard to TNF-α, similar patterns of differences were also observed in the plasma, ileum, and colon levels of TNF-α among the different groups except that 27-OHC treatment did not induce a significant increase in WT mice. However, no significant changes were observed in the IL-10 levels among the different groups (Fig. 19a, b, *P*> 0.05), but ANS treatment could induce significantly increased IL-17 levels in the plasma, ileum, and colon (Fig. 19c, d, *P*< 0.05).

## Discussion

Hypercholesterolemia in midlife is considered to be a modifiable risk factor for the development of AD and dementia [28]. In addition, mice fed a high-cholesterol diet tend to develop memory deficits [29]. Given that cholesterol itself cannot pass through the BBB, it is still unclear how peripheral cholesterol disturbances have a negative impact on cognition [30].

As the most abundant oxysterol and BBB-permeable cholesterol metabolite in the blood, 27-OHC has been reported to induce cognitive impairment. Research from our laboratory suggested that 27-OHC induces neurotoxic effects in the brain in vivo as well as in neuronal cells in vitro, involving mechanisms of neuroinflammation [17], pyroptosis [31], synaptic dysfunction [32], and Aβ deposits [22]. In addition, high plasma levels of 27-OHC were also shown to be a risk factor for mild cognitive impairment [33]. However, the 27-OHC-driven gut dysbiosis, intestinal barrier impairment, and inflammation that triggers cognitive deficits in mice have not been investigated.

The main findings of this study were as follows: (i) 27-OHC induced gut and circulating inflammation and alterations in the gut microbiome and SCFAs, disrupting the intestinal barrier, which may allow the passage of bacterial products and subsequently aggravate the brain amyloid plaque burden and cause cognitive deficits. (ii) Cotreatment with an inhibitor of 27-OHC synthesis, ANS, reduced gut and systemic inflammation and preserved the structure of the intestinal barrier. In conclusion, we have provided new experimental evidence for a critical role of 27-OHC in gut dysbiosis and impairment of intestinal barrier permeability that aggravates inflammation and leads to cognitive deficits and an increased Aβ load in APP/PS1 mice. These results also suggest that pharmacological treatment with ANS may preserve the integrity of the intestinal barrier, alleviate inflammation, and reverse the cognitive deficits induced by 27-OHC.

Cognitive deficits are the main manifestation of AD [34]. In the present study, the data showed that 27-OHC effectively aggravated cognitive deficits in the APP/PS1 mice as shown by the Morris water maze test and the passive avoidance test. Meanwhile, brain Aβ deposition is a hallmark of AD pathogenesis and an important cause of cognitive decline [35]. In this study, we found that 27-OHC treatment could increase amyloid deposition and the plasma and brain levels of Aβ1-40 and Aβ1-42.

A disruption in the gut microbial composition has been reported to be associated with many neurological disorders including AD [36]. Several studies have conducted comparative analyses on the gut microbiota of APP/PS1 mice, a well-established mouse model of AD, compared with WT mice through sequencing of bacterial 16S ribosomal RNA genes. These findings have provided important clues about gut microbiota regulation in regard to combating and preventing AD.

Shen et al. [37] found that the microbiota diversity of APP/PS1 mice was significantly decreased compared with WT mice. We observed that the fecal microbial diversity, as estimated by the Ace, Chao1, and Shannon indices, changed significantly in the cotreatment with 27-OHC/ANS groups as compared with ANS-treated groups, suggesting that 27-OHC treatment could also cause decreased microbiota diversity. In addition, global similarities between bacterial groups were analyzed by β diversity analysis using unweighted UniFrac distance and the findings indicated that the 27-OHC treatment significantly influenced microbiota composition. Therefore, differences in composition coincided with differences in diversity indices.

Further inspection showed that *Roseburia* abundance at the genus level and the fecal levels of some SCFAs were significantly lower in the 27-OHC-treated mice than the control and ANS-treated mice. *Roseburia* is one of the most abundant butyrate-producing bacteria and accounts for 0.9 to 5.0% of the total microbial load [38]. The abundance of *Roseburia* has been reported to be decreased in many intestinal disorders [39] as well as in Parkinson disease [40], indicating the bacterium plays an important role in maintaining the gut microbiome homeostasis by producing SFCAs. Endogenous SCFAs are mainly produced by the gut microbiota (i.e., intestinal commensal bacteria and/or probiotic bacteria) from dietary carbohydrates [41]. Meanwhile, dietary supplementation with some SCFAs, specifically butyrate, has been demonstrated to protect against obesity, hepatic steatosis, and insulin resistance in animal models [42], which are all risk factors for cognitive impairment. In line with the decreased abundance of *Roseburia*, fecal levels of propionate, butyrate, caproate, valerate, and acetate were also significantly decreased in 27-OHC-treated mice. *Roseburia* has also been reported to exert anti-inflammatory effects in in vivo and in vitro models by inducing anti-inflammatory cytokine production and decreasing the production of proinflammatory cytokines [43].

Meanwhile, significant increases in isobutyrate and isovalerate in 27-OHC-treated WT and APP/PS1 mice were also observed. In a randomized crossover study, the fecal concentrations of both isobutyrate and isovalerate were significantly decreased in subjects with metabolic syndrome, including dyslipidemia, insulin sensitivity, and abdominal obesity, in response to dietary fiber intervention [44], indicating that increased cholesterol levels may be linked to elevated isobutyrate and isovalerate. A 3-week supplementation with fructo-oligosaccharides and galacto-oligosaccharides in mice has demonstrated associations between the decreased cecal isobutyrate concentration accompanying reduced pro-inflammatory cytokine levels and depression-like and anxiety-like behaviors [45]. Moreover, Zhuang et al. have found that a significantly higher level of isobutyrate in the feces of constipated people that were also characterized by reduced *Faecalibacterium*, *Ruminococcaceae*, and *Roseburia* abundance [46]. Therefore, these two SCFAs may act as potential mediators of 27-OHC-microbiota-gut-brain crosstalk through inflammation pathways. Therefore, 27-OHC treatment tended to shift the gut microbiota toward profiles that share features with those of AD and inflammatory disorders.

There is growing evidence indicating that cholesterol auto-oxidation products, namely oxysterols, can promote and stimulate intestinal inflammatory diseases with pro-inflammatory properties and by inducing epithelial barrier disturbance. A recent study showed the ability of a combination of dietary auto-oxidation oxysterols to induce the loss of intestinal epithelial layer integrity in monolayers of differentiated CaCo-2 cells [47], with subsequent hyperactivation of pro-inflammatory cytokines matrix metalloproteinases (MMP)-2 and -9 and decreased levels of tight junction proteins including ZO-1, occludin, and junction adhesion molecule-A (JAM-A). Therefore, inflammation could exacerbate barrier dysfunction in a synergistic manner.

For example, treatment with 7-ketocholesterol, an oxygenated cholesterol product present in foodstuffs, reduced epithelial barrier function and diminished IL-10 mRNA expression, an anti-inflammatory cytokine, suggesting oxysterols may contribute to the dysregulation of epithelial barrier function and an inappropriate inflammatory response [48]. Here, we provided in vivo evidence that one of the major enzymatic origin oxysterols, 27-OHC, could also induce a downregulation of tight junction proteins including occludin, claudin 1, claudin 5, ZO-1, and anti-inflammatory cytokine IL-10; upregulation of pro-inflammatory IL-1β; as well as modifications in the intestinal tight junction ultrastructure; pathological changes; and increased levels of plasma d-lactate and DAO; all of which indicated that the intestinal barrier dysfunction became severe after treatment with 27-OHC. Disruption of the intestinal barrier will undoubtedly lead to increased intestinal permeability and systemic exposure to bacterial products such as lipopolysaccharide (LPS) and other luminal endotoxins, pathogens, and antigens, which could trigger systemic inflammatory responses [49].

Accumulated evidence has reported that impaired intestinal barrier integrity is mechanistically involved in the pathological progression of neuroinflammation and cognitive deficits [50]. Gut-blood barrier permeability has also been proposed as an important mechanism for AD development as well [51]. Therefore, barrier function impairment has emerged as a new therapeutic strategy for AD prevention and treatment.

The antagonistic actions of ANS against 27-OHC treatment have been described in our previous studies [21, 22]. In the current study, the administration of ANS reduced the production of systematic and intestinal pro-inflammatory cytokine IL-1β, which mediates the 27-OHC-induced inflammation. We also showed a partial effect of ANS in inhibiting 27-OHC-induced downregulation of tight junction proteins and pathological changes in the ileum and colon as well as in alleviating cognitive deficits and Aβ load, so the anti-inflammatory and antagonistic actions of ANS on cytokines and intestinal barrier permeability may contribute to a partial protective effect against intestinal barrier dysfunction and brain impairment. Taken together, we observed that in animals being cotreated with ANS, the intestinal barrier was preserved at the level of inflammation and certain tight junction proteins. This possible defensive effect of ANS in physiological conditions deserves further investigation.

## Conclusions

In summary, 27-OHC treatment induced dysfunction of the intestinal barrier, characterized by an increased intestinal inflammatory response, loss of tight junction proteins, and gut microbiota and SCFAs dysbiosis, which resulted in elevated systematic inflammation, brain Aβ deposits, and cognitive deficits. Cotreatment with ANS ameliorated the systematic and local inflammatory responses, partially preventing the damage to the tight junction proteins and brain induced by 27-OHC. The deterioration of the intestinal barrier and gut microbiota changes induced by 27-OHC may be some of the mechanisms leading to cognitive deficits. This provides new insight into exploring the neurotoxic effects of 27-OHC and suggests that ANS-based pharmacotherapies, such as a coadjuvant strategy, could be potentially useful to treat brain disorders characterized by 27-OHC overload.

## Data Availability

The datasets during and/or analyzed during the current study available from the corresponding author on reasonable request.

## Abbreviations

27-OHC: 27-Hydroxycholesterol
ANS: Anastrozole
AD: Alzheimer’s disease
DAO: Diamine oxidase activity
Aβ: Amyloid-β
MCI: Mild cognitive impairment
BBB: Blood-brain barrier
CNS: Central nervous system
SERM: Selective estrogen receptor modulator
ER: Estrogen receptor
APP: Amyloid precursor protein
EDTA: Ethylenediaminetetraacetic acid
PBS: Phosphate buffer solution
HE: Hematoxylin and eosin
BSS: Bielschowsky silver stain
TEM: Transmission electron microscope
GAPDH: Glyceraldehyde 3-phosphate dehydrogenase
OUT: Operational taxonomic units
ANOVA: Analysis of variance
LEfSe: Linear discriminant analysis effect size
SPF: Specific pathogen free
WT: Wild type
